# PLAIDOH: a novel method for functional prediction of long non-coding RNAs identifies cancer-specific LncRNA activities

**DOI:** 10.1186/s12864-019-5497-4

**Published:** 2019-02-15

**Authors:** Sarah C. Pyfrom, Hong Luo, Jacqueline E. Payton

**Affiliations:** Department of Pathology and Immunology, Washington University School of Medicine, St. Louis, MO 63110 USA

**Keywords:** Long non-coding RNA, lncRNA, Transcriptional control, *cis-*regulation, Lymphoma, Interactome, RNA-binding protein, Epigenetics

## Abstract

**Background:**

Long non-coding RNAs (lncRNAs) exhibit remarkable cell-type specificity and disease association. LncRNA’s functional versatility includes epigenetic modification, nuclear domain organization, transcriptional control, regulation of RNA splicing and translation, and modulation of protein activity. However, most lncRNAs remain uncharacterized due to a shortage of predictive tools available to guide functional experiments.

**Results:**

To address this gap for lymphoma-associated lncRNAs identified in our studies, we developed a new computational method, Predicting LncRNA Activity through Integrative Data-driven ‘Omics and Heuristics (PLAIDOH), which has several unique features not found in other methods. PLAIDOH integrates transcriptome, subcellular localization, enhancer landscape, genome architecture, chromatin interaction, and RNA-binding (eCLIP) data and generates statistically defined output scores. PLAIDOH’s approach identifies and ranks functional connections between individual lncRNA, coding gene, and protein pairs using enhancer, transcript *cis*-regulatory, and RNA-binding protein interactome scores that predict the relative likelihood of these different lncRNA functions. When applied to ‘omics datasets that we collected from lymphoma patients, or to publicly available cancer (TCGA) or ENCODE datasets, PLAIDOH identified and prioritized well-known lncRNA-target gene regulatory pairs (e.g., HOTAIR and HOX genes, PVT1 and MYC), validated hits in multiple lncRNA-targeted CRISPR screens, and lncRNA-protein binding partners (e.g., NEAT1 and NONO). Importantly, PLAIDOH also identified novel putative functional interactions, including one lymphoma-associated lncRNA based on analysis of data from our human lymphoma study. We validated PLAIDOH’s predictions for this lncRNA using knock-down and knock-out experiments in lymphoma cell models.

**Conclusions:**

Our study demonstrates that we have developed a new method for the prediction and ranking of functional connections between individual lncRNA, coding gene, and protein pairs, which were validated by genetic experiments and comparison to published CRISPR screens. PLAIDOH expedites validation and follow-on mechanistic studies of lncRNAs in any biological system. It is available at https://github.com/sarahpyfrom/PLAIDOH.

**Electronic supplementary material:**

The online version of this article (10.1186/s12864-019-5497-4) contains supplementary material, which is available to authorized users.

## Background

Long non-coding RNAs exhibit remarkable cell type specificity and disease association, yet the vast majority remain uncharacterized. Transcribed pervasively across the genome, they are defined by only two characteristics: length greater than 200 bp and lacking protein coding potential [1]. The diversity of lncRNAs is therefore extremely broad: from a few hundred bases to many kilobases in size; single exon to many exons; present in a wide range of locations ranging from intergenic, intronic, overlapping coding exons, and anti-sense to within coding genes [2]. LncRNAs exhibit much less conservation between species compared to coding genes and structure-function relationships have yet to be defined [3]. A major obstacle to functional discovery is the paucity of established rules and algorithms for functional prediction, and the lack of sequence conservation or homology across species further limits the potential for in silico screening or prioritization for experimental studies [4]. For these reasons, the study of lncRNAs remains challenging.

Despite these challenges, some lncRNAs have been shown to play key roles in a broad range of biological processes via an impressive variety of mechanisms. Reported functions range from *cis-* or trans-regulation of neighboring or distal genes via recruitment or sequestering of epigenetic or transcriptional protein complexes [5, 6]; regulation of chromatin structure and 3D nuclear organization [7–9]; regulation of translation via direct binding to mRNAs [8, 10–12]; and modulation of cellular signaling via protein scaffolding [2, 12]. LncRNAs play roles in diverse essential biological processes, including development (e.g., *XIST, H19*) [6, 13–18], immune response (e.g., *NRON*, *Morrbid*), [5, 12, 19], and oncogenesis (*MALAT1*, *MEG3*) [4, 6, 10, 11, 20]. LncRNAs also have potential clinical utility as disease biomarkers and therapeutic targets, especially in cancer [10, 11].

In pursuit of novel gene regulatory mechanisms that are altered in human Non-Hodgkin Lymphoma (NHL) [21], we identified hundreds of annotated and novel lncRNAs that are significantly altered in primary NHL compared to normal control B cell samples sorted from tonsils and peripheral blood. We next sought to select the most potentially lymphomagenic lncRNAs for targeted, mechanistic experiments. However, prioritization and selection a small number of lncRNAs proved challenging due to the aforementioned paucity of information regarding function. There are a few bioinformatic tools available for lncRNA analysis and functional prediction [22–25]. However, each has disadvantages including: being based entirely on motif predictions; having limited options for customization to end-user priorities; and no integration of lncRNA-associated data into a calculated score that can be ranked. Therefore, we sought to design a new bioinformatic method that would 1) integrate diverse types of -omics data in ways relevant to lncRNA function; 2) be flexible to incorporate user-provided or publicly available datasets in any combination; 3) generate a statistically ranked output; 4) be robust even for smaller datasets, as these are more common and more challenging to analyze; 5) enable users to customize prioritization and ranking metrics to answer specific questions and for selection of top hits for downstream experiments. We named our method PLAIDOH, for Predicting LncRNA Activity through Integrative Data-driven ‘Omics and Heuristics.

PLAIDOH is a new method with modular algorithms that calculate predictive scores based on several different measures of transcriptional regulatory control, protein interaction, and subcellular localization. PLAIDOH integrates transcriptome, subcellular localization, enhancer landscape, genome architecture, chromatin interaction, and RNA-binding data, generating statistically-defined output scores to rank functional connections between individual lncRNA, coding gene, and protein pairs. When applied to ‘omics datasets that we collected from Washington University lymphoma patients [21], or to publicly available cancer (TCGA) [26, 27] or ENCODE datasets [1, 28], PLAIDOH accurately identified and prioritized well-known lncRNA-target gene regulatory pairs (e.g., HOTAIR and HOX genes, PVT1 and MYC) [29–35], positive hits in a CRISPRi lncRNA growth screen [28], and lncRNA-protein binding partners (e.g., NEAT1 and NONO) [36]. Importantly, PLAIDOH also identified novel putative functional interactions, including one lymphoma-associated lncRNA based on analysis of data from our human Non-Hodgkins Lymphoma study [21, 37]. We validated PLAIDOH’s predictions for this lncRNA using knock-down and knock-out experiments in lymphoma cell models. In summary, we show that PLAIDOH fills an important void with a facile method to predict the function of lncRNAs in a variety of systems, and to accelerate validation and follow-on experimental studies to better characterize lncRNAs.

## Results

### Hundreds of LncRNAs are dysregulated in lymphoma and Normal B cells

**Fig. 1:**
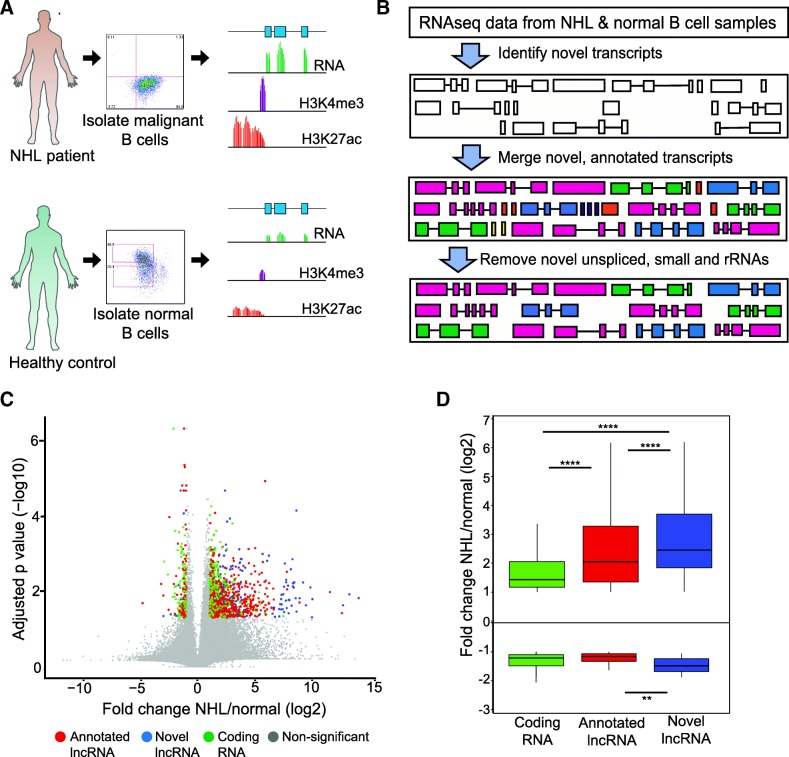
Hundreds of lncRNAs are dysregulated in NHL compared to normal B cells. **a** Schematic depicts collection, flow cytometry purification, and ‘omics profiling of malignant and normal B lymphocytes from NHL patients and healthy volunteers [21]. **b** Diagram of NHL lncRNA discovery pipeline. RNA-seq data was analyzed using a de novo processing pipeline to enable identification of novel transcripts (Cufflinks). Novel RNA transcripts were merged with annotated transcripts (Cuffmerge). **c** Volcano plot highlights lncRNA transcripts with significantly different expression in NHL tumor samples compared to normal B cells (red). Relative expression of lncRNA transcripts shown in log2 fold change expression (FPKM) versus –log10 adjusted *p* value (FDR, Benjamini&Hochberg) for NHL:normal B cells. **d** Data as in C, with different types of lncRNAs highlighted in different colors (red: annotated lncRNAs, blue: intergenic lincRNAs, green: novel (not annotated) lncRNAs

We have previously demonstrated that deregulation of B cell signaling and activation pathways via perturbed enhancer activity and transcription factor expression promotes survival and proliferative pathways and may drive human lymphoma pathogenesis [21, 37]. However, the mechanisms underlying these changes have not been fully characterized. To address this knowledge gap, we sought to identify long non-coding RNAs that may be involved in lymphoma pathogenesis. We built an RNA-seq analysis pipeline designed to discover novel (not previously annotated) long non-coding RNAs, and to quantify expression of all known and novel RNA transcripts in our primary NHL and normal B cell samples (Fig. 1a-b).

Similar to previous reports [1, 38], we found that putative novel single exon lncRNA transcripts are abundant, representing 74% (191,762 of 259,429 unique transcripts detected) (Additional file 1: Figure S1A). However, these novel unspliced transcripts exhibit very low expression (mean FPKM 0.18) compared to novel multi-exon spliced transcripts (mean FPKM 7.3) or annotated (known) single or multi-exon lncRNA transcripts (mean FPKM 10.98) (Additional file 1: Figure S1B). In addition, single exon transcript expression was inconsistently detected across samples: only 10% of transcripts were detected in 10% or more of samples; 80% of novel unspliced transcripts were detected in only 1 sample. In contrast, 60% of annotated lncRNA transcripts were detected in more than 10% of samples, and nearly 90% of protein coding gene transcripts were detected in at least 10% of samples (Additional file 1: Figure S1C). We reasoned that experimental study of lncRNAs with very low expression levels or those detected in < 10% of samples would be challenging, and of questionable relevance to the majority of patients with lymphoma. Moreover, some studies have suggested that spliced, multi-exon lncRNAs may be more likely than unspliced lncRNAs to play defined roles in cellular homeostasis [39]. For these reasons, we focused on multi-exon spliced lncRNAs for subsequent analyses.

We next sought to identify potentially lymphomagenic lncRNAs for targeted mechanistic studies in lymphoma models. Expression in lymphoma compared to normal B cell samples revealed 1464 significantly altered RNAs (log2 fold change > 1, FDR < 0.05). These were distributed among coding mRNA, annotated lncRNA, and novel spliced lncRNA (Fig. 1c).The novel spliced lncRNAs exhibited a larger difference in mean fold change and higher average expression level (Fig. 1d and Additional file 1: Figure S1B). These significantly altered coding genes were enriched in immune response and immune signaling, transcriptional regulation, and cell cycle pathways, consistent with our previous analyses of Follicular Lymphoma [21]. The same enrichment analysis cannot be performed for lncRNAs because databases of lncRNA functions and pathways do not yet exist in standardized forms, and therefore lncRNA gene symbols are generally not included in the output from ontology and pathway annotation tools.

### Overview of PLAIDOH pipeline and modular algorithms

**Fig. 2:**
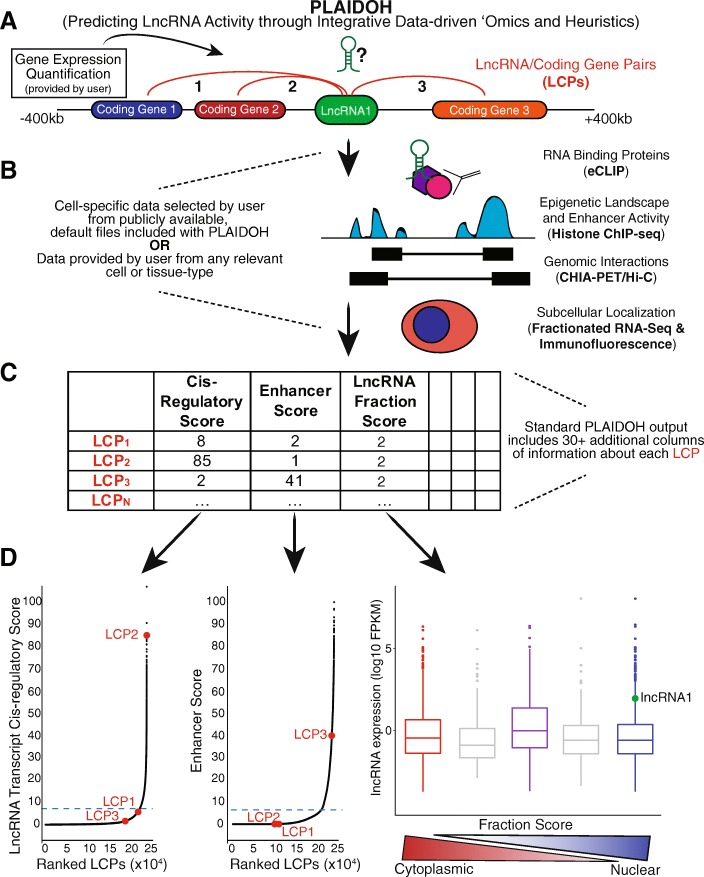
An overview of the PLAIDOH pipeline and algorithm output. **a** Schematic of the single, input file required by PLAIDOH to identify all possible lncRNA/Coding gene Pairs (LCPs) in the user’s dataset. **b** Overview of the datasets that are used by PLAIDOH to annotate lncRNAs and predict activity based on genomic and epigenomic context. **c** Abridged example of the primary PLAIDOH output table, showing the three scores PLAIDOH calculates for each LCP as well as the 30+ additional columns of valuable information about the lncRNA and coding gene in each LCP. **d** Examples of graphs output by PLAIDOH as part of its standard run settings. The three LCPs and lncRNA1 diagrammed in **a** are highlighted in red and green, respectively

An overview of the PLAIDOH pipeline and selected example output graphs are shown in Fig. 2. LncRNAs have been categorized in many ways: including by function, localization, size, and site of transcription [2, 39]. Functionally, a simple separation can be made between lncRNAs that impact gene transcription, those that act post-transcriptionally to alter protein expression, and those that act in other cellular pathways (Fig. 2a). Our goal was to develop a method robust enough for smaller datasets such as those generated by a single lab, rather than a large consortium (e.g., TCGA). Thus, we focused our pipeline and algorithms on experimental data, rather than on motifs or binding predictions. We first focused on modulation of gene expression (*cis-*regulatory), because these are generally thought to be the largest group of lncRNAs [2, 39], and because publicly available RNA-seq datasets provide gene regulatory input *and* output data (i.e., expression) upon which to train a predictive method. We reasoned that a predictive tool could be further improved by the inclusion of transcriptional control mechanisms, such as data from epigenetic profiles, enhancer and super-enhancer landscapes, and genome architecture. For *trans-*regulatory lncRNAs, we incorporated RNA-binding protein interactome and subcellular localization data. We incorporated these normalized and statistical data with regression analyses to generate predictive scores for each potential function and protein or gene partner (Fig. 2b, Additional file 2: Table S1). These statistically ranked output scores can be tailored for prioritization of top hits based on user preference. Thus, PLAIDOH is a simple, user-friendly informatic pipeline and set of algorithms that integrates genome, transcriptome, and interactome datasets and calculates three predictive scores based on several different measures of transcriptional regulatory control, protein interaction, and cellular pathway.

### Expression correlation identifies potential regulatory relationships

**Fig. 3:**
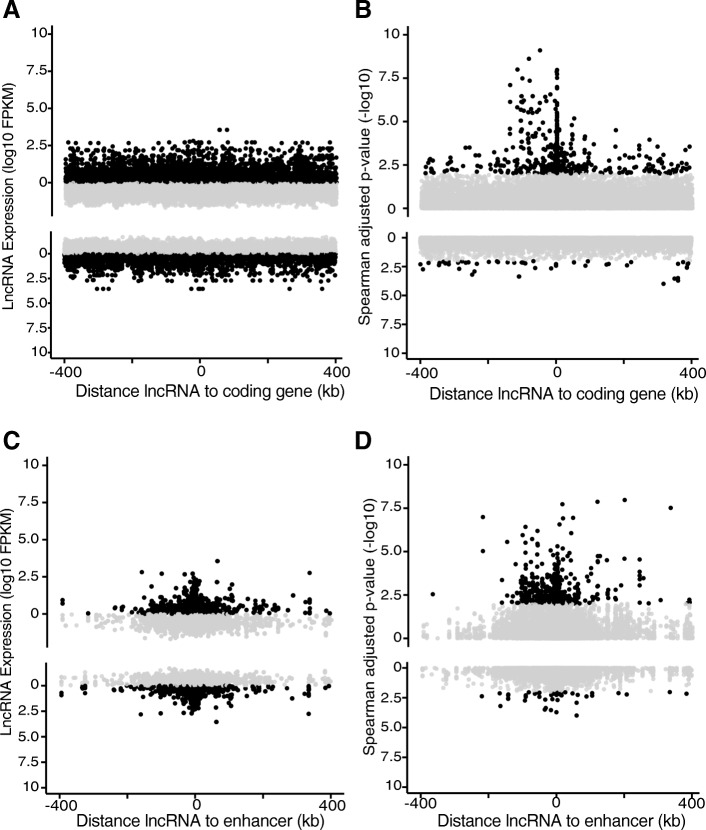
PLAIDOH reveals global patterns of LCP co-expression. LncRNA expression (log10 FPKM) (**a** & **c**) or LCP correlation (−log10 Spearman adjusted *p*-value) (**b** & **d**), are plotted relative to genomic distance from each lncRNA to a coding gene (**a** & **b**) or the nearest enhancer (**c** & **d**) within 400 kb regions flanking the lncRNA. LCPs with positive Spearman correlation coefficients (rho) are plotted in the upper half of each plot; those with negative Spearman correlation coefficients (rho) are plotted in the lower half. Black points highlight LCPs with adjusted Spearman *p*-values < 0.05 or FPKM > 1

We posited that an initial measure of a lncRNA’s effect on a coding gene’s expression is correlation of expression between the two transcripts, which may be positive or negative. We sought to calculate the most statistically robust associations given that most experimental -omics datasets, including our NHL/normal B cell data, are relatively small and/or heterogeneous (e.g., derived from outbred humans). Therefore, we focused on local coding genes, defined as those within 400 kb flanking a lncRNA. We chose this total distance (800 kb) because it is the size of an average topologically associated domain (TAD) [40]. Each lncRNA had a median of six coding genes within 400 kb flanks (range: 0–68), though these numbers varied by reference annotation used for each dataset and the number of lncRNAs defined therein. There was no significant association of lncRNA expression level with distance to coding genes (Fig. 3a, Additional file 3: Figure S2A). Moreover, separating lncRNAs into groups defined by location relative to coding genes (overlapping, anti-sense, or distal) revealed that overlapping and antisense lncRNAs did not have higher expression than distal lncRNAs (Additional file 3: Figure S2B).

Next, we calculated the Spearman correlation coefficient and adjusted *p* value for each lncRNA-coding gene pair (LCP) within 400 kb on either side of each lncRNA across all samples. We observed that a greater number of LCPs located close together (< 5 kb) had significant expression correlation (adj *p* < 0.05) compared to those located further apart (Fig. 3b, Additional file 3: Figure S2C). As expected, the expression correlation of LCPs containing antisense or coding-gene overlapping lncRNAs had more significant *p* values as compared to distal, though a subset of these distal LCPs showed a highly significant correlation, suggesting they may have a regulatory role (Additional file 3: Figure S2D).

### Enhancer activity, proximity, and chromatin interaction associate with higher LCP correlations

A large proportion of lncRNAs are known to overlap with enhancer elements and some have been shown to be involved with enhancer-mediated transcriptional control [1, 2, 5]. Location of a lncRNA gene within 1 kb of a FANTOM enhancer has recently been shown to be significantly associated with lncRNAs that modified cell growth upon CRISPRi targeting [28]. Consistent with these and other reports, we observed an increase in the average expression level of lncRNAs located close to FANTOM enhancers, for both positively and negatively correlated LCPs (Fig. 3c). We also observed that a greater number of LCPs located close to FANTOM enhancers (< 100 kb) had significant expression correlation (adj *p* < 0.05) compared to those located further away from enhancers (Fig. 3d).

Super-enhancers (SE) are dense clusters of very active enhancers that drive the transcription of genes involved in cell identity [41]. LncRNAs associated with SE have been linked to key developmental processes and master transcription factors in hematopoietic, cardiac, and embryonic stem cells [42–46]. Therefore, we reasoned that lncRNA expression level may be correlated with co-localization with SEs. However, we found no enrichment of lncRNAs located within SEs compared to conventional enhancers. In fact, conventional enhancers harbored significantly more lncRNAs per kb than SEs (Additional file 4: Figure S3A). Moreover, we found no difference in expression between lncRNAs within SEs compared to those in conventional enhancers, but those not in enhancers had significantly higher expression (Additional file 4: Figure S3B). We next separated lncRNAs into those that are intergenic (do not overlap a coding gene) and those that are intragenic (at least 1 base pair overlap with a coding gene). This separation demonstrates that lncRNAs that are not located within any enhancer, but do overlap a coding gene, exhibited significantly higher expression levels compared to any other group. This group of intragenic, non-enhancer lncRNAs includes lncRNAs entirely encompassed within introns as well as anti-sense lncRNAs (Additional file 4: Figure S3C).

We next reasoned that quantitative measurement of regulatory activity would provide additional metrics by which to rank lncRNAs by transcriptional control capacity. To discriminate transcriptional regulation by enhancers overlapping lncRNA gene loci from regulation mediated by lncRNA transcripts themselves, we incorporated chromatin landscape and genome architecture metrics for the region surrounding each lncRNA locus (400 kb). For enhancer activity, we included chromatin immunoprecipitation sequencing data (ChIP-seq) for H3K4me1 and H3K27ac, which are histone modifications associated with all enhancers or active enhancers, respectively, and for H3K4Me3, which marks active promoters. The *p* values (−log10) for ChIP-seq peaks overlapping lncRNA genes were used as a measure of normalized enhancer activity [47, 48]. As expected, for the activating H3K4me3 and H3K27ac marks, higher activity was associated with increased lncRNA expression, especially for H3K4me3, while little effect on expression was observed for the H3K4me1 modification, which marks both active and inactive enhancers (Additional file 4: Figure S3D). In contrast, there was little to no effect of epigenetic activity on the expression correlation between LCPs (Additional file 4: Figure S3E). Taken together, these results suggest that enhancer activity, as well as proximity to enhancers and coding genes, contribute to lncRNA expression level but do not provide sufficient information to predict lncRNA function, and therefore additional information was needed.

Enhancers often regulate gene expression by direct physical interaction with target gene promoters via transcriptional control proteins (activators, repressors, transcription factors, epigenetic modifiers). This interactive conformation is referred to as chromatin looping or genome architecture and can be measured by Chromatin Interaction Analysis by Paired-End Tag Sequencing (ChIA-PET) [40, 49–52]. ChIA-PET interaction scores [49, 52] reflect the relative frequency of the interaction between two genomic fragments, bound by an immunoprecipitated protein. As shown in Additional file 5: Figure S4A and B, there is a relative enrichment of POL2RA ChIA-positive LCPs with higher ChIA scores among those that are located more closely in genomic space, as would be expected. In addition, there are a greater number of LCPs with significant expression correlations (Spearman adjusted *p*-values < 0.05) located close together Additional file 5: Figure S4C).

To modulate transcription of another gene, a lncRNA must be located in the nucleus. On the other hand, post-transcriptional regulation or functions unrelated to gene expression (e.g., regulation of mRNA translation, modulation of protein signaling activity, etc.) require cytoplasmic localization. Therefore, PLAIDOH incorporates data from subcellular fractionation RNA-seq [1] to calculate nuclear:cytoplasmic ratios of total RNA transcript for each lncRNA. We selected this data source because it provided subcellular localization for a larger number of lncRNAs than another database [53] and because the data is derived from the same cell types used in our analyses (ENCODE cell lines, B cells). We observed a trend toward higher correlation coefficient values with higher nuclear:cytoplasmic ratios, though again only for the positively correlated LCPs (Additional file 5: Figure S4D). These results underscore that multiple mechanisms contribute to transcriptional control, and therefore PLAIDOH integrates data from each of them to make functional predictions for individual lncRNAs.

### PLAIDOH distinguishes poly- versus mono-genic regulatory patterns

**Fig. 4:**
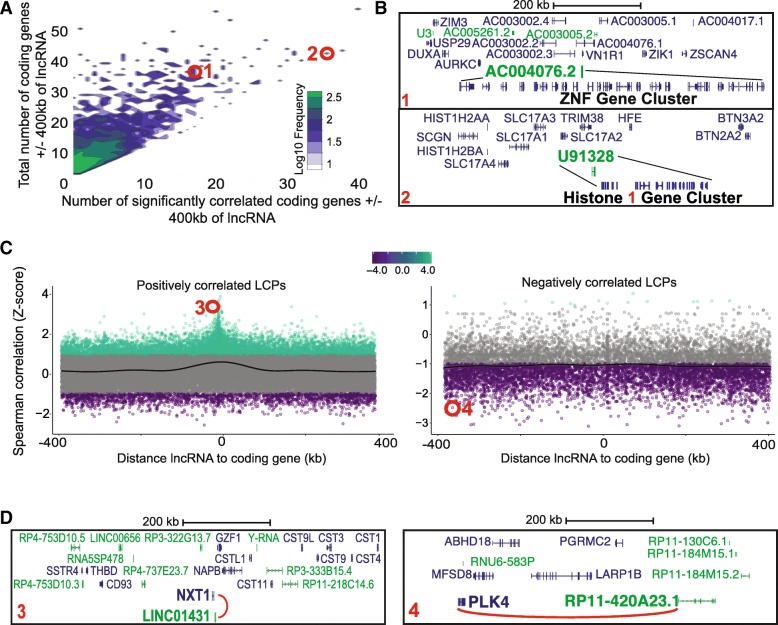
PLAIDOH ranks lncRNAs by number and fraction of correlated coding genes. **a** Contour plot shows the frequency of significant LCPs numbers as a function of the number of all possible coding gene pairs for each lncRNA. Color indicates increasing log10 frequency of LCPs at each x,y data point (white-blue-green). Highlighted in red are two LCPs in which single lncRNAs are each highly-correlated with large clusters of coding genes. **b** Genomic maps of the two LCPs shown in **a**. **c** Z-Scores of LCP correlation coefficients plotted by distance between each lncRNA and coding gene pair; positively correlated LCPs are plotted in the left panel and negatively correlated LCPs are in the right panel. Highlighted in red are LCPs in which single lncRNAs are correlated with only one coding gene. **d** Genomic maps of the LCPs shown in **c**

In evaluating the potential of lncRNAs for transcriptional regulatory control, we observed that some lncRNAs demonstrated distinct patterns of correlative expression relative to the coding genes within their local genomic environment. We sought to differentiate lncRNAs that correlate with all, some, or only one gene within 400 kb up- or down-stream, since these patterns may indicate different transcriptional control mechanisms: generalized contribution to transcriptional activity in a locus versus regulation of a specific gene. To segregate these distinct patterns, we devised two new data visualizations and applied them to data from acute myeloid leukemia (AML) from TCGA [26]. First, we created a 3-dimensional frequency matrix surface plot to show the frequency of LCPs for a specific number of significantly correlated coding genes versus the total coding genes within 400 kb flanking the lncRNA. This contour plot effectively segregates lncRNAs that are significantly correlated with large numbers and high proportions of neighboring coding genes (Fig. 4a). We highlight two such lncRNA pairs that are correlated with large clusters of coding genes (Fig. 4b). The expression of *AC004076.2* is significantly and positively correlated with a large cluster of zinc finger KRAB repressor domain-containing proteins. This correlation is in contrast to the other lncRNA genes in this locus, suggesting a specific transcriptional control relationship between this lncRNA and this cluster of zinc-finger transcriptional repressors. The second example is lncRNA *U91328*, whose expression is significantly and negatively correlated with a cluster of Histone H1 genes. Histone H1 linker histone proteins interact with DNA between nucleosomes and are involved in chromatin condensation, nucleosome remodeling, regulation of transcription, and DNA replication. Histone H1 genes are mutated in some hematopoietic cancers; they exhibit altered and heterogeneous expression within tumors; and silencing of these genes is associated with maintenance of self-renewal capacity in tumor cells [54–57]. These two lncRNAs exemplify the ability of PLAIDOH analysis and the 3-D frequency matrix plot to visually highlight lncRNAs that correlate with and may contribute to the transcriptional control of a large number and/or large proportion of coding genes.

Next, we sought to identify individual lncRNAs that are significantly more correlated with a single coding gene compared to all others in the 400 kb flanking regions. We identified such “outliers” by calculating the Z-scores of the Spearman correlation of each LCP for a given lncRNA, to identify those with the correlation that most deviates from the rest of the LCPs. Plotting these by genomic distance from the lncRNA visually highlights those with the highest Z-scores (Fig. 4c); two examples are shown in Fig. 4d. Among many coding genes in its 400 kb neighborhood, *LINC01431* is significantly correlated only with nearby *NXT1*. The two genes are transcribed from the opposite strands such that their 3′ ends are within 2 kb, but they do not overlap or share a promoter/TSS. *NXT1* codes for an essential nuclear transport factor involved in the export of mRNA and small RNA molecules from the nucleus [58, 59]. Strong correlation with a single coding gene is not limited to those located close together, however. Another lncRNA, *RP11-420A23.1*, is significantly correlated only with *PLK4*, which is located 391 kb away. *PLK4* codes for Polo-like Kinase 4, one of a family of serine-threonine kinases that regulates centriole duplication during mitosis and is a target of inhibitors currently in Phase III clinical trials for AML [60, 61]. Thus, this feature of PLAIDOH analysis can identify highly specific and differential correlative relationships between lncRNAs and coding genes that may indicate transcriptional control of a single coding gene.

### PLAIDOH identifies Cancer-type specific and recurrent LCPs

**Fig. 5:**
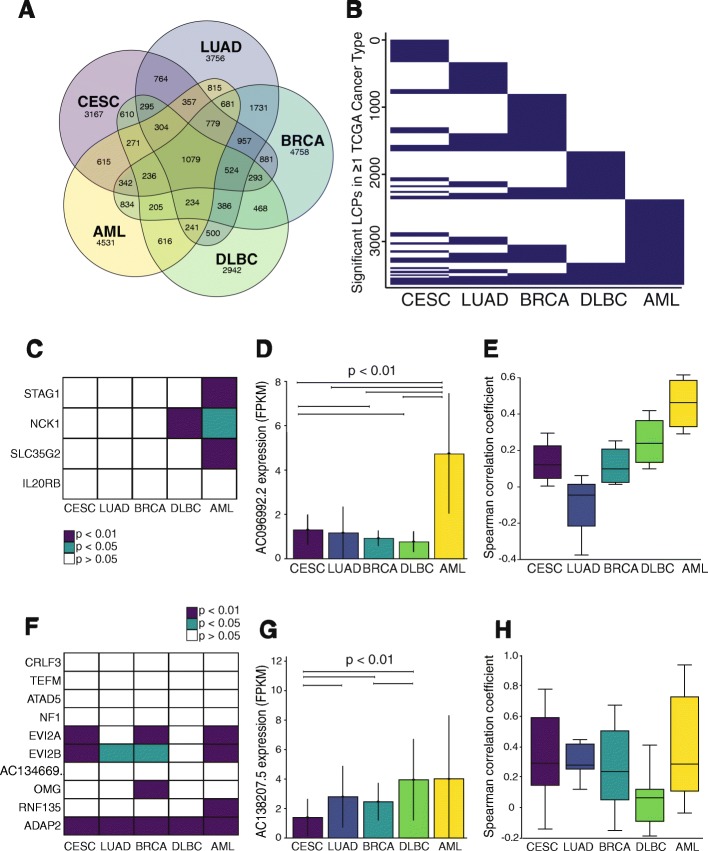
LncRNAs demonstrate common or cancer-type specific correlation profiles. **a** Venn diagram shows the number of significant LCPs shared or unique among five TCGA cancer types. Significant = Spearman correlation adj *p* < 0.05 for LCP expression. **b** Binary heatmap shows the pattern of correlation significance for LCPs across TCGA cancer types. Spearman adj p < 0.05 (purple); *p* > 0.01 (white). **c** Heatmap of LCP Spearman correlation p-values for expression of *AC096992.2* and each of the genes within 400 kb. Spearman adj *p* < 0.01 (purple); p < 0.05 (blue); *p* ≥ 0.05 (white). **d** Bar graph shows expression of *AC096992.2* in TCGA cancer types. **e** Box plot shows Spearman correlation coefficients (rho) for expression of *AC096992.2* and all genes within 400 kb flanking. **f** Heatmap of LCP Spearman correlation p-values for expression of *AC138207.5* and each of the genes within 400 kb flanking. Colors as in C. **g** Bar graph shows expression of *AC138207.5* in TCGA cancer types. **h** Box plot shows Spearman correlation coefficients (rho) for expression of *AC138207.5* and all genes within 400 kb flanking

Thus far, we have only applied PLAIDOH to monotypic datasets (all one cell or one tumor type) or to small datasets from a few cell lines. We next sought to evaluate whether similar regulatory patterns were observed for lncRNAs across diverse human cell types and from large sample sets. In terms of number of primary human samples, the largest and most diverse -omics study to date is The Cancer Genome Atlas (TCGA) [26, 27]. We compared five different cancer types from TCGA, including epithelial and hematopoietic lineage (acute myeloid leukemia, AML; breast cancer, BRCA; cervical cancer, CESC; diffuse large B cell lymphoma, DLBC; and lung adenocarcinoma, LUAD). The Venn diagram and heatmap in Fig. 5a-b show that these cancers have common and distinct sets of significant positively or negatively correlated lncRNA-coding gene pairs. The majority of these (56%) are significant in only one cancer type (cancer-specific) and less than 2 % (1.8%) are significant in all five cancer types (cancer-recurrent). Negatively correlated, significant LCPs comprise a much smaller subset that is almost exclusively cancer-type specific (Additional file 6: Figure S5A-B). These results likely reflect the known cell-type specificity of lncRNA expression patterns, and may indicate that the observed specificity is due to the role of lncRNAs in transcriptional regulation.

In Fig. 5c-h, we highlight examples of cancer-specific and cancer-recurrent LCPs. The cancer-specific pattern is exemplified in the *STAG1* locus, in which three of four genes within the 800 kb window are significantly correlated with *AC096992.2* in AML (Fig. 5c). STAG1 is a ubiquitously expressed component of the cohesin complex that stabilizes topologically associating domain (TAD) boundaries with CTCF [62]. Mutations in *STAG1* and other cohesin complex genes are recurrent in AML [63]; cohesin dysfunction leads to alterations in gene regulation that contribute to leukemogenesis [64]. NCK1 is adaptor protein that is downstream of receptor tyrosine kinase and STAT3 signaling; both *NCK1* and the other correlated gene, *SLC35G2*, may be involved in colorectal cancer pathogenesis [65, 66]. *AC096992.2* exhibits much higher expression in AML (Fig. 5d) and is more highly correlated with neighboring genes in AML compared to the other cancer types (Fig. 5e). This effect was not due to generally higher expression of all of the neighboring genes in AML compared to the other cancer types (Additional file 6: Figure S5C), and levels of nearby active and enhancer-associated histone marks H3K27ac and H3K4me1 were not consistently higher in AML compared to the other tumors (Additional file 6: Figure S5D). These results suggest that lncRNA *AC096992.2* itself may play a *cis-*regulatory role in the transcription of multiple neighboring genes.

The cancer-recurrent pattern for an LCP is exemplified in the *EVI2/ADAP2* 17q11.2 locus (Fig. 5f-h), which is associated with Neurofibromatosis type 1 microdeletion syndrome, a severe phenotype with overgrowth and increased neurofibromas [67, 68]. Of the 9 coding genes and one other lncRNA in this locus, expression of *AC138207.5* is significantly correlated with *ADAP2* in all five cancer types, and with *EVI2A/B* in most of the five cancer types (Fig. 5f-g). ADAP2 is a ubiquitously expressed GTPase activating protein for ARF6 that acts as a scaffold in innate immune and membrane inositol phosphate signaling pathways [69–71]. *EVI2A* and *EVI2B* are named for being ecotropic viral integration sites; *EVI2B* expression is altered in some types of AML and is a regulator of hematopoietic stem/progenitor cell and myeloid differentiation [72]. These results suggest that lncRNA *AC138207.5*, which exhibits robust expression across the five cancer types (Fig. 5h), may play a *cis-*regulatory role in the transcription of multiple neighboring genes in diverse cancer types. We note that this feature of PLAIDOH could also be used for customized comparisons between user-provided experimental groups (e.g., tumor:normal, treated:control, developmental stages, or time points).

### PLAIDOH segregates LncRNAs by putative transcriptional control mechanism using enhancer and LncRNA transcript Cis-regulatory scores

Up to this point, we have evaluated the contribution of discrete transcriptional regulatory mechanisms that may be part of lncRNA function. Our next step was to integrate these distinct measures into a unified predictive model. Therefore, we devised two scores designed to reflect the relative evidence of enhancer- or lncRNA transcript-mediated (“*cis*”) transcriptional control. The Enhancer score incorporates data from measures of transcriptional control by enhancer regulatory elements: overlapping enhancer-associated H3K4me1 and active enhancer-associated H3K27ac levels for each lncRNA, and the presence and relative strength of chromatin looping between the lncRNA and the coding gene pair as measured by ChIA-PET [49, 52] (see Methods). The lncRNA transcript *cis-*regulatory score gives greater weight to data associated with the lncRNA transcript itself. This score is calculated from the adjusted *p*-value of the Spearman LCP correlation, the level of overlapping promoter-associated H3K4me3 activity, and the fraction of lncRNA transcript localized in the nucleus (see Methods).

**Fig. 6:**
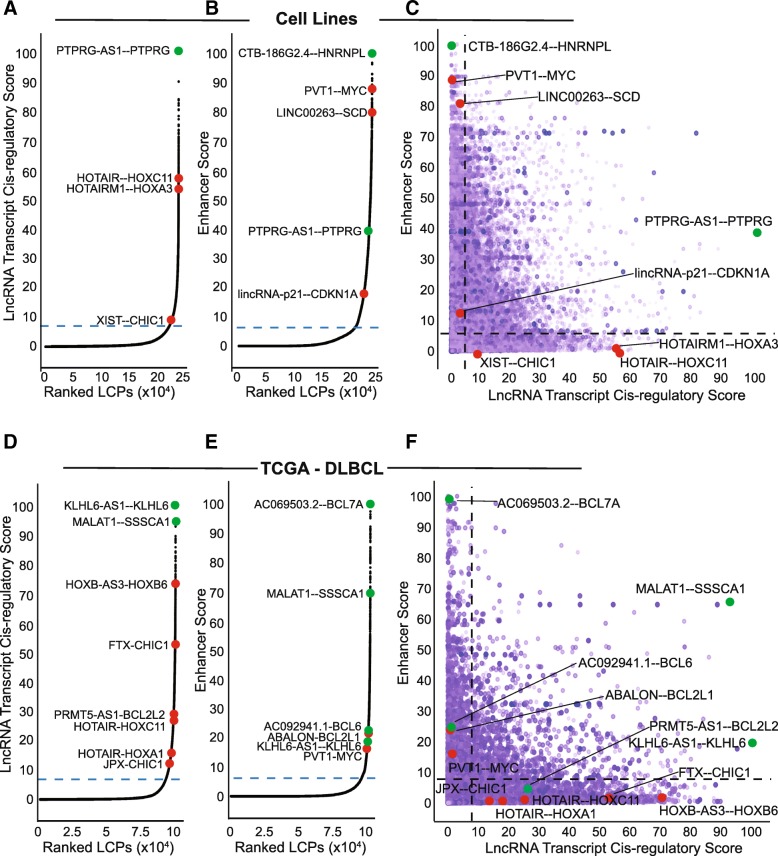
PLAIDOH ranks LCPs by likely transcriptional regulatory mechanism, inferred from Enhancer and LncRNA *Cis-*regulatory Scores. **a**-**f** Plots show LCPs from ENCODE cell lines (**a**-**c**) or TCGA DLBC samples (**d**-**f**). **a** & **d** Plots show LCPs ranked by increasing LncRNA Transcript *Cis-*regulatory Scores. Red points are known *cis-*acting lncRNAs; in green are novel LCPs with the highest scores and/or containing known lymphoma oncogenes. **b** & **e** As in A&D, but ranked by increasing Enhancer Scores. Highlighted in red are known enhancer-associated lncRNAs; in green are novel LCPs with the highest scores and/or containing known lymphoma oncogenes. **c** & **f** XY plots show Enhancer versus LncRNA Transcript *Cis-*regulatory Scores segregating LCPs. Dotted lines in **a**-**f** reflect score cut-offs based on the geometric inflection points calculated from the data in **a**, **b**, **d** & **e**. Red and green data points are from **a** & **b** (for **c**), or **d** & **e** (for **f**)

To validate the enhancer and lncRNA transcript *cis-*regulatory predictive scores, we calculated these scores for all LCPs across several different ENCODE cell lines (K562, HeLa, U87, MCF7, MDA-MB-231) and in five cancer types from TCGA (AML, BRCA, CESC, DLBC, LUAD). Ranking the LCPs from least to greatest for each score revealed an inflection point in the distribution of scores. We geometrically defined the inflection point as the cut-off for high predictive scores (see Methods). Ranked plots from ENCODE and TCGA datasets show several lncRNAs and their known coding gene *cis-*regulatory targets among the highest lncRNA transcript and enhancer *cis-*regulatory scores (Fig. 6 and Additional file 7: Figure S6, red points), including *HOTAIR* and *HOTAIRM1* and *HOX* genes [29–34], *XIST/FTX/JPX* and *CHIC1* [73–76], *lincRNA-p21*(*PANDAR*) and *CDKN1A(p21, 77].* Also ranked highly by PLAIDOH’s enhancer *cis-*regulatory score are *LINC00263* and its paired coding gene *SCD* (Fig. 6b). *LINC00263* was a validated hit in a CRISPRi cell growth screen in ENCODE cell lines using dCas9-KRAB to epigenetically repress lncRNAs [28]. Similarly, *PVT1* has a very highly ranked enhancer *cis-*regulatory score, was also a CRISPRi hit in ENCODE breast cancer cell lines [28], and demonstrated to inhibit *MYC* expression in *cis* via promoter competition for common enhancer elements [35]. Many of these LCPs were also ranked highly by enhancer *cis-*regulatory score in multiple TCGA cancer types (Fig. 6e and Additional file 7: Figure S6). The role of lncRNAs in the pathogenesis of Non-Hodgkin B cell Lymphoma (NHL) has not been extensively evaluated. In samples from TCGA DLBCL, which are a type of NHL, we identified several lymphoma oncogenes within LCPs with high enhancer or transcript *cis-*regulatory scores, including anti-apoptotic factors *BCL2L2* (BCL-W) and *BCL2L1* (BCL-XL), *BCL6*, and *BCL7A*, suggesting that the paired lncRNAs may play a role in the transcription of these lymphoma-associated genes (Fig. 6d-f, green points). These and other top-scoring LCPs in cancer cell lines and other TCGA cancer types have not been previously reported, and may represent novel lncRNA cis-regulatory relationships (Fig. 6 and Additional file 7: Figure S6, green points).

When both scores are plotted together on an XY graph, the score cutoffs define 4 quadrants, with distinct potential functional interactions for the LCPs within them (Fig. 6c & f, and Additional file 7: Figure S6C,F,I,L). For all datasets, the vast majority of LCPs (84.2–89.5%) cluster in quadrant one, with low scores for both enhancer and lncRNA transcript *cis-*regulatory function and the lowest median expression levels for lncRNA and coding genes (0.05–0.23 and 1.36–3.36 FPKM, respectively). These results suggest that, for the majority of potential lncRNA coding gene pairs, the coding gene’s transcription is not controlled by either the lncRNA or its overlapping enhancer. Quadrant two contains far fewer LCPs (3.43–8.01%), and is defined by high enhancer scores, low transcript *cis-*regulatory scores, and relatively higher median lncRNA and coding gene transcript levels (0.25–0.65 and 5.02–6.59 FPKM, respectively). Location of an LCP in quadrant two suggests that transcriptional control of the paired coding gene may be mediated through the lncRNA’s overlapping enhancer element, and that lncRNA transcripts here may function via epigenetic mechanisms, e.g. recruitment of chromatin modifiers and/or facilitating chromatin looping. Indeed, several lncRNAs known to act via such epigenetic *cis-*regulatory control of coding gene partners are located in quadrant two (*LINC00263—SCD*, *PVT1—MYC*, and *PANDAR/lincRNA-p21--CDKN1A* Figs. 6c & f) [28, 35, 77]. Quadrant three is defined by high scores for both enhancer and lncRNA transcript *cis-*regulatory control, contains the fewest LCPs (1.35–3.06%), has the highest median lncRNA expression and nearly the highest coding gene expression (0.43–1.34 and 5.26–7.23 FPKM, respectively). Thus, quadrant three may represent the strongest regulatory relationships between lncRNAs and the paired coding genes, with evidence for both enhancer-mediated and transcript-mediated *cis-*regulatory mechanisms, resulting in relatively higher expression of both groups (e.g., PTPRG-AS1—PTPRG, KLHL6-AS1—KLHL6 Figs. 6c & f. Finally, 2.8–6.0% of LCPs are located in quadrant four, which is defined by a high lncRNA transcript *cis-*regulatory score, a low enhancer score, and relatively lower median expression levels for both lncRNAs and coding genes (0.3–1.1 and 2.9–4.5 FPKM, respectively). Location in this quadrant may indicate that, independent of enhancer augmentation, lncRNA *cis-*regulatory control may be possible, albeit with relatively lower target coding gene expression levels. The predictive power of assignment to quadrant four is validated by lncRNAs known to regulate expression of coding gene pairs via transcript-mediated *cis-*regulatory mechanisms, including *HOTAIR—HOX* genes [29–34] and *XIST/JPX/FTX--CHIC1* [73–76]. Thus, PLAIDOH integrates global measures of transcriptional control to calculate scores that predict the relative likelihood of distinct *cis-*regulatory control mechanisms, which are consistent with published reports for many lncRNAs as shown here.

### PLAIDOH compares favorably to lncRNA CRISPR screens and other lncRNA analytical tools

To further test how well PLAIDOH’s predictions of transcriptional regulatory function coincide with lncRNAs’ experimentally-defined function, we compared PLAIDOH’s Enhancer and lncRNA Transcript cis-regulatory scores with the results of several published CRISPR lncRNA screens. Each screen used a different CRISPR approach: knock-down (CRISPR-KRAB), knock-out, and activating (CRISPR-CaLR) [28, 78, 79]. The knock-down and KO screens used readouts of cell proliferation/growth as readouts, while the activating CRISPR screen used susceptibility to AraC (alkylating chemotherapeutic agent). Thus, these screens identified lncRNAs that affect cell growth (or cell growth in the setting of AraC treatment), while PLAIDOH identifies lncRNAs that modulate the expression of other genes (LCPs) or interact with RNA-binding proteins, regardless of their effect on cell growth. Additional file 8: Figure S7A shows that, overall, PLAIDOH’s Enhancer and lncRNA Transcript cis-regulatory scores are significantly higher in hits compared to non-hits for the CRISPR-KRAB screen. The significant difference in both enhancer and transcript-mediated regulatory scores is not surprising given that the KRAB knock-down is mediated via suppressive epigenetic marks targeted to the lncRNA region, and therefore would likely suppress both lncRNA transcription and enhancer activity [28]. Overall, these results suggest that lncRNAs with higher PLAIDOH scores may be more likely to have functional cellular effects when suppressed.

Next, we compared to validated lncRNA hits from the CRISPR KO [78] and activating screens [79] (Additional file 8: Figure S7B&C). Of the validated lncRNAs with sufficient data for PLAIDOH analysis, the majority had PLAIDOH Enhancer or Transcript Cis-Regulatory scores above the cutoffs: 11/15 (73.3%) from the CRISPRa screen and 9/14 (64.3%) from the CRISPR KO screen. Further confirmation of PLAIDOH’s prioritization scores is evidenced by several validated anti-sense (AS) lncRNAs in the CRISPRa screen. For all but one (RERG-AS1), PLAIDOH’s scores were above the cutoffs for Enhancer or Transcript cis-regulation (Quadrant 2 or 4, respectively), and the highest scores were for the matched coding gene. The low (Quadrant 1) score for RERG-AS1--RERG was due to low expression levels in both the lncRNA and the coding gene (0.0036 and 0.014 FPKM) and lack of overlapping active chromatin marks, though there is a high scoring CHIA interaction. Taken together, these results suggest that the lncRNAs’ effects on cell growth may be mediated through modulation of the expression of nearby coding genes, which were identified by PLAIDOH.

During PLAIDOH’s development, we compared it to other available lncRNA analysis methods and tools. Because lncRNAs are still a relatively new class of genes, many of the available tools were limited to processing and analyzing RNA-seq data, identifying and annotating known and novel lncRNA transcripts [23, 80, 81]. In contrast, we designed PLAIDOH to address the functional knowledge gap for lncRNAs by analyzing transcriptome, epigenome, 3D-genome interaction, cellular localization, and RBP-lncRNA interactome data to make functional predictions for lncRNAs. Thus, here we will compare PLAIDOH to other informatic tools or methods that perform similar analyses. One of these, called lncRNA-screen, comprises a pipeline that processes RNA-seq data to identify known and novel lncRNAs, and integrates ChIP-seq and Hi-C data [24]. Like PLAIDOH, lncRNA-screen outputs a table of data related to each lncRNA. However, the table contains discrete data only; there is no integration into a predictive or rankable score as PLAIDOH calculates. Also, like PLAIDOH, lncRNA-screen generates graphical output, but in the form of heatmaps and genome snapshots, which are useful for a global overview or for visualization of a specific locus. Again, these graphical outputs do not display prioritize lncRNAs by individual metrics or integrated scores, nor do they compare potential functions of individual lncRNAs side-by-side. By contrast, PLAIDOH generates two types of cis-regulatory score for individual lncRNA-coding gene pairs, as well as analyzing and integrating RNA-binding protein interactome data (described further below). Direct comparison of a specific example, lncRNA *CTD-2006C1.2*, shows that lncRNA-screen and PLAIDOH both identified *ZNF44* and *ZNH439* as potential cis-regulatory targets. However, PLAIDOH provides individual cis-regulatory scores for each LCP (18.7 and 26.6, respectively) and also identifies another coding gene, *ZNF20*, with a higher cis-regulatory score (27.7) and stronger expression correlation (rho = 0.849, adj *p* < 2.2 × 10^-16).

Another group developed an analytical approach that combined methods to predict lncRNA regulatory networks [22]. The Pan-cancer analysis of the tumors in TCGA integrated transcriptome and eCLIP data with transcription factor and lncRNA binding motifs, to predict lncRNA regulatory networks and categories of lncRNA function (transcriptional, post-transcriptional, or both). Unlike PLAIDOH, the Pan-cancer method does not incorporate epigenome data, which would provide direct evidence of regulatory activity near or overlapping lncRNAs. The Pan-cancer method predicts many regulatory targets for individual lncRNAs, and these targets can be located anywhere in the genome. PLAIDOH also identifies multiple targets of individual lncRNAs but focuses on 800 kb genomic windows for transcriptional regulatory relationships and provides rankable scores for each pair to enable prioritization. Because of these differences in the outputs of the Pan-cancer analysis and PLAIDOH, we focused our comparison on lncRNAs ranked highly by the former, and, in some cases, validated by siRNA knock-down [22]. Of 43 lncRNAs predicted to regulate gene expression in TCGA cancers by Pan-cancer, 31 had high PLAIDOH Enhancer and/or lncRNA transcript cis-regulatory scores (72.1%, Additional file 8: Figure S7D). These concordant results demonstrate that, while the Pan-cancer method and PLAIDOH incorporate different approaches, there is substantial overlap in highly ranked lncRNAs.

Taken together, comparison of PLAIDOH to lncRNA CRISPR screens or to other analytical methods or tools demonstrates that PLAIDOH’s predictive transcriptional regulatory scores are corroborated by these orthogonal approaches. Moreover, PLAIDOH has some unique features that provide the user with functionality not yet found in other tools/methods.

### PLAIDOH integrates biological metrics to stratify LncRNA-protein Interactomes

**Fig. 7:**
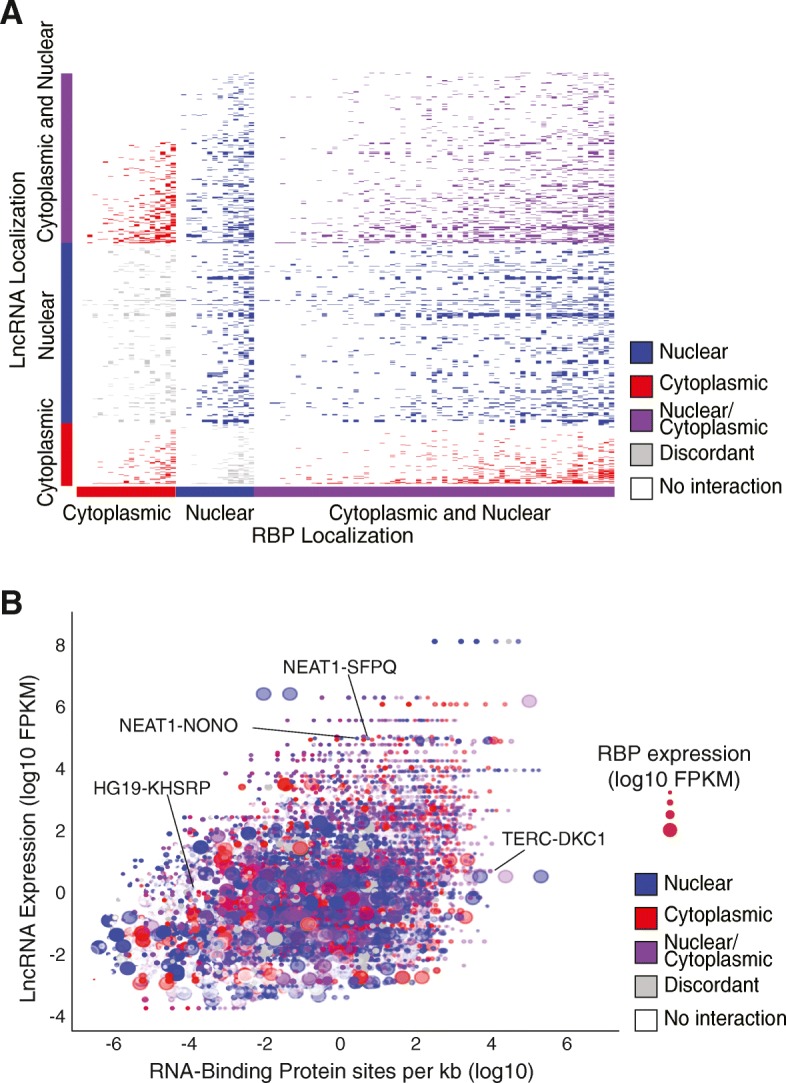
PLAIDOH ranks lncRNAs using biological and experimental data from RNA binding protein interaction. **a** Interaction matrix of lncRNAs and RNA Binding Proteins. Binding events of concordantly localized lncRNAs and RBPs are colored by subcellular localization to the nucleus (blue), cytoplasm (red) or both nucleus and cytoplasm (purple). Discordantly-localized interactions are colored grey. No evidence of binding is white. **b** Plot shows lncRNA expression versus RBP binding-site density per kilobase of RNA transcript for each lncRNA/RBP interaction shown in panel **a**. Data point size is scaled to RBP expression level and subcellular localization interactions are colored to match panel **a**. Labeled dots highlight previously published and validated binding of RBP/lncRNA pairs

Having defined scores to predict lncRNA *cis-*regulatory transcriptional mechanisms, we next turned to incorporating data measuring of other potential lncRNA functions. A major category of function not yet addressed by PLAIDOH is modulation of protein or pathway activity via direct binding of proteins or protein complexes [2, 39, 82]. To identify and rank these potential interactions, PLAIDOH integrates data from RNA-binding protein (RBP) immunoprecipitation sequencing studies (eCLIP-seq) in which the relative strength and significance of the RBP-lncRNA interaction is given a score based on normalized read depth and specificity controls [82]; for this analysis PLAIDOH required a maximum interaction score (see Methods). For corollary confirmatory biological data, PLAIDOH compares subcellular localization of the potentially interacting lncRNA and RBP, as determined by subcellular fraction RNA-seq, Western blot, and immunofluorescence [1, 82], and outputs a coefficient of co-localization. Figure 7a shows this interactome localization analysis as an interaction matrix of lncRNAs and RBPs, in which interaction and co-localization are color-coded, segregated, and sorted. Each row represents a lncRNA and each column an RBP. Grey blocks indicate an eCLIP interaction but the lncRNA and RBP pair are not co-localized; red, blue, or purple indicate co-localization of the pair in the cytoplasm, nucleus, or in both fractions, respectively. By sheer number and by percentage of significant binding interactions, the greatest fractions are comprised of lncRNA-RBP pairs with cytoplasmic and/or nuclear co-localization. By comparing the number of colored blocks for each, one can appreciate the number of significantly interacting RBPs per lncRNA (and vice versa). Most of the RBPs interact with multiple lncRNAs, which is not surprising given that the target proteins were selected based on known RNA-binding function. Many of the lncRNAs also interact with multiple RBPs, but PLAIDOH’s analysis and the interaction matrix shown in Fig. 7a enable filtering or selection of lncRNA-RBP pairs based on cellular co-localization and specificity of interaction.

To enable prioritization of lncRNAs, we next integrated additional quantitative data, including lncRNA and RBP expression levels, number of RBPs bound per lncRNA (a measure of specificity), and number of RBP binding sites per kb of spliced lncRNA (a potential measure of binding strength, normalized for lncRNA size) for more granular ranking of lncRNA-RBP pairs. Figure 7b demonstrates how this approach can stratify lncRNA – RBP pairs by lncRNA and RBP expression, RBP binding site density, and co-localization in the same subcellular fraction. The effectiveness of this method is confirmed by accurate stratification of several lncRNA-RBP partners, including highly expressed lncRNA NEAT1 with two different paraspeckle proteins (NONO and SFPQ) [36, 83], all co-localized in the nucleus; the moderately expressed telomerase lncRNA TERC with a relatively high number of RBP sites per kb for the telomerase complex-associated protein, dyskerin (DKC1), which are co-localized in the nucleus [84]; and the moderately expressed lncRNA HG19, which has relative few RBP binding sites per kb and acts as a molecular scaffold to facilitate effective association of K homology-type splicing regulatory protein (KHSRP) with labile transcripts [85], both of which co-localize in the cytoplasm. These results validate PLAIDOH’s approach, and demonstrate that these integrative analyses enable filtering and prioritization of lncRNAs based on relevant biological and experimental data related to lncRNA - RBP interactomes.

To further validate PLAIDOH’s lncRNA-RBP interactome analyses, we compared to CRISPR screen hits that had validated growth phenotypes but low PLAIDOH cis-regulatory scores (Additional file 8: Figure S7B). RBP-interactome analysis shows that some of these lncRNAs may function via interactions with RNA-binding proteins in non-transcriptional pathways (e.g., RNAi, ribosome biogenesis, splicing) as indicated by PLAIDOH analysis of RBP binding, expression levels, and subcellular localization of these lncRNA-protein partners (Additional file 9: Figure S8).

### Validation of PLAIDOH’s predictions for a lymphoma-associated lncRNA

**Fig. 8:**
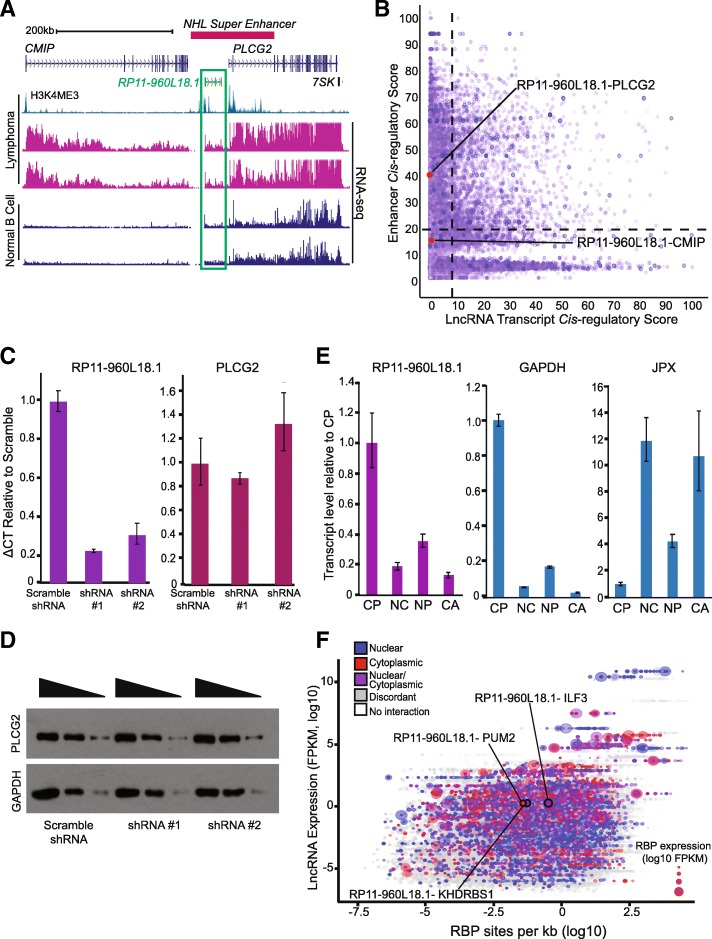
Validation of PLAIDOH’s functional predictions for a lncRNA highly expressed in human NHL. **a** UCSC Genome browser view of HK4me3 ChIP-seq (NHL) and RNA-seq (NHL, normal B cells) for the *RP11-960 L18.1* locus. **b** XY plot shows Enhancer versus LncRNA Transcript *Cis-*regulatory Scores in primary NHL samples, highlighted are *RP11-960 L18.1* and the two most proximal coding genes. **c** Expression of *PLCG2* and *RP11-960 L18.1* measured by qRT-PCR in HBL1 lymphoma B cell line treated with scramble or one of two *RP11-960 L18.1* shRNAs. **d** Western Blot for PLCG2 or GAPDH in HBL1 cells treated with scramble or one of two *RP11-960 L18.1* shRNAs. Triangles indicate relative number of cells loaded on the gel. **e** Subcellular localization of RNA transcripts determined by cell-fractionation of control (WT) HBL1 cells followed by qRT-PCR (CP: cytoplasm, NC: nuclear, NP: nucleoplasm, CA: chromatin-associated). **f** Plot shows lncRNA expression versus log_10_ RBP binding-site density per kilobase of RNA transcript for each lncRNA/RBP interaction, highlighted are RBPs that bind *RP11-960 L18.1*. Data point size is scaled to RBP expression level and subcellular localization interactions are colored as in Fig. 7

We developed PLAIDOH to prioritize and help us to select a small number of human NHL-associated lncRNAs to move forward for focused experimental studies. One of these is highly and recurrently expressed in lymphoma compared to normal B cells: *RP11-960 L18.1* (Fig. 8a). It is located upstream of the gene that codes for PLCG2, a phospholipase C family enzyme specific for B lymphocytes that is activated by B cell receptor (BCR)-associated kinases upon antigen-mediated BCR stimulation. By converting PIP2 to IP3 and diacylglycerol (DAG), PLCG2 increases intracellular Ca2+ levels and activates B cell signaling pathways, including NF-kB, NFAT, and Ras, and their downstream gene targets [86]. Located within an NHL super-enhancer, *RP11-960 L18.1* has a high enhancer *cis-*regulatory score with *PLCG2* (42.1, quadrant two); while its score with another neighboring gene, *CMIP,* was below the cutoff (18.0, quadrant one). The lncRNA transcript cis-regulatory scores for both genes are also below the cutoff (2.9 for *PLCG2*, 1.5 for *CMIP*)) (Fig. 8a-b). These results suggest that transcriptional regulation of *PLCG2* may be through enhancer-mediated mechanisms rather than via direct activity of the *RP11-960 L18.1* transcript itself. To test the role of the lncRNA transcript itself, we used shRNA knock-down and CRISPR knock-out of *RP11-960 L18.1* in lymphoma B cell lines that highly express RP11-960 L18.1 (HBL1, U2932). Using two different shRNAs or CRISPR-Cas9 mediated deletion of the first two exons of *RP11-960 L18.1*, we successfully decreased expression of RP11-960 L18.1 by 70–95%. However, neither mRNA nor protein levels of *PLCG2* were affected (Fig. 8c-d, Additional file 10: Figure S9A-B). These results suggest that the RP11-960 L18.1 transcript itself does not substantially modulate the expression of PLCG2 in B cells, however the overlapping enhancer region that was left largely intact may have a *cis-*regulatory role in control of PLCG2 transcription.

We next sought to determine where RP11-960 L18.1 transcript is localized, given that cytoplasmic localization is associated with non-transcriptional lncRNA functions. Subcellular fraction RNA-seq data showed that a greater fraction of RP11-960 L18.1 transcript was located in the cytoplasm [1]. Since the RNA-seq data was from normal B cells, we confirmed it in lymphoma B cell lines (HBL1, U2932) by subcellular fractionation and qRT-PCR of cytoplasmic, nuclear, nucleoplasm, and chromatin-associated fractions. As shown in Fig. 8e, RP11-960 L18.1 is predominantly localized to the cytoplasm. For comparison, control mRNA GAPDH is predominantly localized to the cytoplasm and the CTCF-associated noncoding RNA JPX is enriched in the nuclear and chromatin-associated fractions [75, 87]. We next evaluated the RBP interactome of RP11-960 L18.1 using PLAIDOH and our primary human NHL RNA-seq data integrated with eCLIP-seq data [21, 82]. This approach revealed that RP11-960 L18.1 interacts with a small number of RBPs, including ILF3/NF90, KHDRBS1/SAM68, and PUM2, all of which are highly expressed in the NHL samples (FPKM 14.6–26.4) and also localize to the cytoplasm (Fig. 8f). Supporting these results, RBP motif scans showed 9 KHDRBS1/SAM68 motifs in the RP11-960 L18.1 transcript sequence, all within 300 bp of the 3′ end of the molecule [88]. The evidence for binding to both SAM68 and PUM2 is intriguing, since interaction with both of these RBPs was recently reported for NORAD, noncoding RNA activated by DNA damage, another predominantly cytoplasmic lncRNA. SAM68 and PUM2 promote genome stability and cell cycle progression, contributing to cell proliferation [89–91]. Data from the eCLIP studies also show that RP11-960 L18.1 has 11 binding sites for ILF3/NF90, which is highly expressed in the NHL samples (average 26.4 FPKM). NF90 is a dsRNA binding protein that forms complexes with several other proteins in different contexts to regulate gene expression, stabilize mRNAs, and promote cell growth and proliferation in embryonic stem cells as well as cancer cells [92–94]. Taken together, these results suggest that RP11-960 L18.1 is a cytoplasmic lncRNA that may function via interactions with cytoplasmic protein(s) to promote the growth and proliferation of B lymphocytes.

In summary, our studies describe how our methods can identify lncRNAs that are significantly dysregulated in human lymphoma, as compared to normal B cells. To predict their function and prioritize these dysregulated lncRNAs for experimental studies, we developed PLAIDOH, an integrative, data-driven approach to accelerate lncRNA prioritization for functional studies, a heretofore highly manual process. We demonstrate that PLAIDOH accurately predicted and prioritized known lncRNA- coding gene or -RNA binding protein interactions and, importantly, predicted a novel lncRNA function that was confirmed by experimental studies in lymphoma cell models. Thus, PLAIDOH fills an unmet need in the study of lncRNAs: a flexible, accessible method for prediction and ranking of functional roles and specific targets of lncRNAs, expediting the transition to focused experimental follow-up studies.

## Discussion

LncRNAs have emerged as a new frontier of biological molecules with enormous potential for increasing our understanding of disease mechanisms and providing new therapeutic targets. CRISPRi screens and focused mechanistic studies have begun to assign function to a small number of lncRNAs, but the vast majority remain functionally uncharacterized. A major obstacle to functional discovery is the relative lack of established rules or algorithms for functional prediction and prioritization for experimental studies. After identifying hundreds of lncRNAs significantly altered in human lymphoma samples, we encountered this obstacle in attempting to prioritize the lncRNAs for focused experimental studies. Therefore, we developed a bioinformatic analysis pipeline with modular algorithms that integrates diverse types of ‘omics datasets (transcriptome, epigenome, 3D-genome, protein interactome) and generates statistically ranked output scores based on several different measures of activity level, transcriptional regulatory control, and protein interaction for selection of top hits for targeted, mechanistic experiments.

PLAIDOH has several unique features that provide the user with functionality not yet found in other tools. PLAIDOH uses only experimental data in its algorithms, which is in contrast to other approaches that rely partially or wholly on binding or motif predictions [22, 95]. We chose to focus on experimental data because the results of binding prediction algorithms have not been extensively validated and their inclusion vastly increases the number of potential lncRNA interactions, which decreases statistical power for analyses of smaller and/or heterogeneous datasets, such as our NHL patient samples. For transcriptional control analyses, PLAIDOH focuses on coding genes within 400 kb flanking the lncRNA, though we would note that this genome window is customizable. We chose this approach because most lncRNAs with transcriptional regulatory function characterized to date act in *cis* [2, 11, 39]. Unlike most other methods [23, 24, 80], PLAIDOH integrates multiple measures of transcriptional control to differentiate enhancer-mediated and lncRNA transcript-mediated *cis-*regulatory mechanisms. These measures include ChIP-seq for histone modifications (H3K4me3, H3K27ac, and H3K4me1), which enable quantitative measurement of regulatory element activity [48], and ChIA-PET, which quantitatively measures the genomic interaction of lncRNAs with target gene loci [49, 52]. By calculating independent scores for overlapping enhancer activity and lncRNA transcript *cis-*regulatory activity, PLAIDOH successfully distinguishes these distinct mechanisms of transcriptional control, as demonstrated by identifying known lncRNA-target coding gene interactions such as *PVT1*--*MYC*, *HOTAIR*--*HOX* genes, and *lincRNA-p21(PANDAR)—CDKN1A* [29–34, 77].

LncRNAs also act in *trans* in a range of cellular processes, including mRNA splicing, protein translation, and cellular signaling via direct binding to other RNAs or by acting as scaffolds or guides for proteins and protein complexes [2, 11, 96]. Prediction of these functional classes is the most challenging since most ‘omics data (e.g., RNA-seq) does not capture these interactions. Indeed, while binding prediction, co-expression, or network analysis methods may indicate apparent relationships between the lncRNA and target RNAs or proteins, these are indirect measures at best, and their large number of potential interactions may lead to decreased statistical power for smaller datasets. We therefore incorporated eCLIP-seq data for lncRNA – protein interactome analyses of ENCODE cell lines. These RBP immunoprecipitation studies were extensively validated by shRNA knockdown and controlled for antibody specificity; RBP subcellular localization was determined using the validated antibodies [82]. Integration of eCLIP-seq data with corollary lncRNA expression and localization data enabled prioritization by co-localization, binding specificity, and expression, identifying both literature-supported and novel lncRNA-RBP interactions. Using only eCLIP-seq data for PLAIDOH’s protein interactome input does have the disadvantage of selection bias: only lncRNAs that bind to proteins selected for immunoprecipitation will be assayed, and new lncRNA binding proteins will not be identified. As new eCLIP-seq (or other RBP-lncRNA interaction) datasets become available, these can be easily included in PLAIDOH analyses.

We designed PLAIDOH to be accessible and customizable for experimental labs with limited computational resources and time. PLAIDOH’s software and dependencies are limited to the installation package (available at GitHub [97]) and R [98], which is free, available in graphical user interface form (R Studio), and supported by many online forums and help websites [99]. Because users will have diverse questions to ask of their datasets, we designed PLAIDOH to be customizable as to the data sources and types incorporated, parameters included for ranking, and lncRNA features or mechanisms of action that are prioritized. Users have the option to add their own data, or publicly available data, or a combination, and compare experimental groups. Users may choose to include or exclude nearly any of PLAIDOH’s parameters for mechanistic prediction, since there are few required inputs for PLAIDOH to run successfully. In addition, users can customize the parameters that are included, or not, in the ranking, depending on their experimental design, scientific question, and/or desired downstream study.

The feature that most distinguishes PLAIDOH from other lncRNA analysis tools/methods is that it addresses the most challenging step in ‘omics studies: selecting a small number of “top hits” for focused mechanistic studies. This step is particularly challenging in the study of lncRNAs since there is so little known about their function and essentially no established ontologies. In other lncRNA prediction methods, ranking is either unavailable or based on only one aspect of lncRNA activity (e.g., expression, genomic location) [22–24, 100]. In contrast, PLAIDOH incorporates several measures of lncRNA activity, transcriptional control, and protein interaction to output integrated scores and rankable metrics. This integrated approach is essential for distinguishing among potential mechanisms of action for a given lncRNA, rather than merely ranking on individual data types, and is attainable only when quantitative data is integrated into a scoring system.

Our goal in developing PLAIDOH was to accelerate the laborious process of prioritizing and selecting lymphoma-associated lncRNAs for downstream experimental studies. Here we validated PLAIDOH’s functional predictions for RP11-960 L18.1, confirming that it is not a *cis-*regulatory lncRNA for PLCG2, but rather likely acts in the cytoplasm by interacting with one or more RBPs (NF90, KHDRBS1, PUM2) to promote the growth of lymphoma cells.

## Conclusions

In summary, PLAIDOH provides a flexible, accessible method for the prediction and ranking of functional connections between individual lncRNA, coding gene, and protein pairs. PLAIDOH fills an important void with a facile method to facilitate the study of lncRNAs in a variety of systems, from normal development to cancer. Our method enables prioritization and ranking based on any parameter, function, or interaction of interest to the user, to expedite the transition to focused experimental studies of lncRNAs. We expect that PLAIDOH will accelerate validation and follow-on mechanistic studies to better characterize lncRNAs, which will ultimately provide new insights into the role of these enigmatic molecules in normal and pathogenic cellular processes.

## Methods

### User-generated files and basic PLAIDOH usage

PLAIDOH requires a single user-generated input file, in addition to the publicly available default annotation files that come with PALIDOH. The input file must have columns in the order shown in Table 1 and the first line must begin with a “#”.

**Table 1:** Example PLAIDOH input table. Example header and first two lines of the modified bedfile required from the user as an input file

#CHR	START	STOP	NAME	TYPE	SAMPLE_1_	SAMPLE_N_
chr1	112	256	DHX9	protein_coding	0.675	5.89
chr1	778	4334	AC00896.1	lncRNA	89	4
chr1	334	566	RP9911.3	antisense_rna	8.3	0.33

CHR must be the chromosome for the transcript on that line. The start and stop coordinates may be any region of the transcripts the user wishes to study (ie. TSS to end of transcript), but PLAIDOH will only consider within the boundaries provided by the input file. The NAME category may be either a gene name or an ENSEMBL identifier. The TYPE category may be any identifier the user chooses but PLAIDOH will ONLY provide annotation and predictions for transcripts labeled “protein_coding, “lncRNA” or antisense_rna” all other designations will be filtered into the “misc” output file. The user may include as many samples as they choose and utilize any calculation for expression (e.g., FPKM, CPM, microarray probe count etc.). The input should be sorted by CHR and START coordinate using sort -k1,1 -k2,2n.

A detailed overview of the PLAIDOH output file can be found in Additional file 2: Table S1 and instructions for running PLAIDOH.pl can be found at: www.github.com/sarahpyfrom/PLAIDOH.

### Visualizing trends in lncRNA concordance and expression

For illustrating trends specific to either positively- or negatively-correlated pairs of lncRNAs and proteins we devised plots by the correlation of the lncRNA and protein coding gene, positive Spearman correlation coefficients (rho) values are plotted above the central line and negative Spearman correlation coefficients (rho) values are plotted below. This approach was used in Fig. 3 and Additional file 4: Figure S3.

### Identifying mono- and poly-correlated lncRNAs

Using the PLAIDOH output table generated from TCGA-AML data, the total number of coding genes and the number of coding genes significantly correlated (Adjusted *p* < 0.01) within +/− 400 kb of each lncRNA was calculated. A frequency matrix was plotted with total protein numbers on the x axis and the significantly correlated protein numbers on the y axis. The frequency of each combination of total protein number/number significantly correlated proteins was calculated and plotted on the matrix from all lncRNAs in the dataset. Additionally, a Z-score of all correlations for each lncRNA was calculated and plotted, such that coding genes, which are greater than one standard deviation more or less correlated with a given lncRNA, would be colored purple or green in the resulting graph.

### Cross-Cancer Fidelity of LCPs

For pan-cancer analysis, RNA-seq expression data from TCGA datasets (LUAD, BRCA, CESC, DLBC, AML) were run through PLAIDOH, which automatically calculates correlation values for each LCP. Significantly correlated LCPs conserved across one or more TCGA datasets were identified. Significance was defined as an adjusted spearman *p*-value < 0.05.

### PLAIDOH enhancer and transcript Cis-regulatory output scores

The LncRNA Transcript *Cis-*regulatory score is calculated as follows:

$$ {10}^{\ast}\mathrm{abs}{\left(\left(-\log \left(\left(\mathrm{ADJUSTED}\_\mathrm{SPEARMAN}+0.001\right)\right)\right)\right)}^{\ast }\ \left({\left(\mathrm{H}3\mathrm{K}4\mathrm{ME}3+0.1\right)}^{\ast }\ \left(\mathrm{FRACTION}+0.01\right)\right) $$

The Enhancer score is calculated as follows:

$$ \left(\left(\left(\mathrm{H}3\mathrm{K}4\mathrm{ME}1+1\right)\right)+\left(\ \mathrm{H}3\mathrm{K}27\mathrm{AC}+1\right)\right)\ast \left(1+\left(\mathrm{ChIA}-\mathrm{PET}\ \mathrm{score}\right)/100\right) $$

All underlined components of the above calculations are described in more detail in Additional file 2: Table S1. The cutoffs for PLAIDOH-calculated Enhancer and LncRNA Transcript C*is-*regulatory scores were calculated by plotting ranked scores for each lncRNA/coding gene pair and finding the geometric inflection point, defined as the point at which the linear regression line crossed the ranked scores line. Linear regression lines were calculated using the geom_smooth command from the R ggplot2 package: geom_smooth(method = “lm”, se = FALSE, formula = y~x). Both scores were ranked for graphing output using the following formula: rank (SCORE, na.last = FALSE, ties.method = “random”). All scores were scaled to 100 for graphing in figures.

### RBP binding of lncRNAs

PLAIDOH output was used to create a binding matrix for each RBP to each lncRNA. An RBP was considered to bind a lncRNA if both the K562 and HepG2 eCLIP assays showed binding of the RBP to the lncRNA (score of 1000) OR if both replicates of either cell line showed binding. If an RBP does not show evidence of binding a lncRNA, the overlapping region is given a score of 0 on the matrix.

### Sub-cellular localization of lncRNAs and RBPs

Sub-cellular localization of lncRNAs was determined using GM12878 RNA-seq from ENCODE. Nuclear and Cytoplasmic protein-coding and non-coding FPKMs were downloaded from hg38-aligned RNA-seq. Chromosomal coordinates were then lifted over to hg19 using UCSC’s liftover tool. The total number of reads for each transcript was calculated as the sum of fragments per kb per million reads from each sub-cellular dataset and the percent of the total FPKM was calculated for both the Nuclear and Cytoplasmic datasets. For later analysis, is determined to be “Nuclear” if 70% of the normalized fragments are in the nuclear RNA-seq compartment, “Cytoplasmic” if 70% of the reads are in the cytoplasmic compartment and “Nuclear and cytoplasmic” if between 30 and 70% of the reads are in the nucleus. RBP sub-cellular localization is determined by Immunofluorescence as described in Sundararaman et al., 2016 [82]. For each possible lncRNA/RBP combination a “Localization concordance score” was calculated. If there is no evidence that the lncRNA and RBP interact based on the ENCODE eCLIP data, the pair is given a score of 0. If an RBP binds a given lncRNA and the sub-cellular localization of both the lncRNA and corresponding RBP are both determined to be Nuclear or both are Cytoplasmic, the pair is given a score of 2 or − 2, respectively. If the lncRNA is primarily Nuclear and the RBP does not have a Nuclear localization by Immunofluorescence (or vice versa), the overlapping region is given a score of 1 or − 1. If both the RBP and lncRNA are considered to be present in both the nucleus and cytoplasm (as described above) the pair is given a score of 3.

### Cell culture, Knock-out and Knock-down

HBL1 and U2932 cells were cultured in RPMI complete. CRISPR knock out of RP11-960 L18.1 in U2932 cells was performed as in [37] using target sequences below. HBL1 cells were infected with RP11-960 L18.1 shRNA in pMLP-GFP lentiviral vector purchased from transOMIC technologies. Cells were grown in RPMI complete plus 1μg/ml puro to select for cells successfully infected with lentivirus. Cells were harvested after 10 days of selection for western blot and qPCR. shRNA sequences are as follows (PASSENGER loop GUIDE):

- CRISPR/Cas9 Upstream Target Sequence: GTACGAAACCTCCCCGCGG
- CRISPR/Cas9 Downstream Target Sequence: AGGTAGGAGGAACGCGCTC
- shRNA #1: 5’ ACAGGTCATTCTTCTGCTCTAAtagtgaagccacagatgtaTTAGAGCAGAAGAATGACCTGG 3’
- shRNA #4: 5’ CCACAGGGAAGAATGACTTCAAtagtgaagccacagatgtaTTGAAGTCATTCTTCCCTGTGA 3′

### Western blot

Whole cells were lysed in RIPA buffer supplemented with Roche protease inhibitors, an equal volume of loading dye was added and lysates were boiled for 10 min. The equivalent of 0.13, 0.067 and 0.03 million cells were loaded for cells infected with a control shRNA and each RP11-960 L18.1-targeted shRNAs. After transfer to nitrocellulose paper, blots were incubated in primary antibodies for PLCG2 (Santa Cruz sc-407) and GAPDH (abcam ab9485) at a concentration of 1:1000 overnight at 4C. Donkey Anti-rabbit secondary antibody from GE Healthcare UK Limited (NA9340V) was used at a concentration of 1:10,000 and imaged using Pierce ECL Western Blotting Substrate (32106).

### Cellular fractionation and qRT-PCR

Sub-cellular fractionation of cytoplasm, nuclear total, nucleoplasm and chromatin for RNA isolation was performed as described in in [101]. cDNA was made from whole cells and cell fractions using the High Capacity RNA-to-cDNA Kit from ThermoFisher (4387406). qRT-PCR was performed using primers specific for PLCG2, RP11-960 L18.1, JPX and GAPDH and the SYBR Green Real-Time PCR Master Mix. Sub-cellular Fraction delta Ct values were calculated relative to the Ct values of the cytoplasmic fraction and whole cell post-shRNA delta-Cts were calculated relative to GAPDH levels.

PLCG2 For: TCAATCCGTCCATGCCTCAG

PLCG2 Rev.: CCTCGACGTAGTTGGATGGG

RP11-960 L18.1 For: GTCACACAGCCAACTTGCG

RP11-960 L18.1 Rev.: AGCCTCTATCTGCTTACGTGC

JPX For: GACACTGGTGCTTTCCTGGG

JPX Rev.: TTGTACCACCGTCATCAGGC

GAPDH For: ACCCACTCCTCCACCTTTGAC

GAPDH Rev.: TGTTGCTGTAGCCAAATTCGTT

### WU NHL and Normal B cell De novo RNA-seq analysis pipeline

As described in Koues et al., 2015 [21], briefly: RNA-seq libraries were prepared from rRNA-depleted samples (Ribo-Zero, Epicentre) using TruSeq RNA Sample kits with indexed adaptors (Illumina), pooled (3 libraries), and subjected to 100 bp paired-end sequencing using an Illumina HiSeq2000. RNA-Seq data were aligned to the transcriptome and reference genome (build GRCh37/hg19) with TopHat [102]. RNA-seq data from were processed using Cufflinks, a transcript assembling software package, with settings designed to identify both novel and annotated transcripts. Annotated transcripts were compiled from several sources (ENCODE, ENSEMBLE, see below). A master GTF was created to summarize and merge all transcripts detected across samples. This master GTF was then used to analyze all samples to quantitate expression level of each transcript. In this way, transcript isoform, detection (+/−), and normalized expression level (FPKM) was determined for each sample for all transcripts, to compare expression patterns across sample types. We used CPAT to exclude transcripts with coding potential [103].

### TCGA

TCGA data [26] was downloaded from https://portal.gdc.cancer.gov/. Five datasets were used: LUAD (Lung Adenocarcinoma), BRCA (Breast Invasive Carcinoma), CESC (Cervical Squamous Cell Carcinoma and Endocervical Adenocarcinoma), AML (Acute Myeloid Leukemia) and DLBC (Lymphoid Neoplasm Diffuse Large B-cell Lymphoma). Gene Expression Quantification (RNA-seq) aligned to hg38 was acquired from all tumor types and chromosomal positions were converted to hg19 using the UCSC liftOver tool (http://genome.ucsc.edu/cgi-bin/hgLiftOver). For PLAIDOH analyses, all 48 samples from the DLBC data were compiled into a single input file, and 48 samples were randomly selected from each of the other four cancer sets (LUAD, BRCA, CESC and AML) to create cancer-specific input files with identical transcript annotations.

### Cell line gene expression

Expression of lncRNAs and coding genes from multiple cell lines (K562, U87, HeLa, HEK293T, MCF7, MDA-MB-231 and iPSCs) were graciously provided upon request from [28].

All default input data files were curated from publicly available resources and modified to fit specific file formats as outlined in the PLAIDOH documentation (will be submitted along with the script on github). All default data sources are outlined in the table below:

### Default ‘omics data sources

All default input data files were curated from publicly available resources and modified to fit specific file formats as outlined in the PLAIDOH documentation (www.github.com/sarahpyfrom/PLAIDOH). All default data sources are described in Table 2.

**Table 2:** Data sources for each PLAIDOH default file. Data names, sources and descriptions for all of the metrics utilized by PLAIDOH to annotate lncRNA and gene function

Data source	Data Type	Description	URL
Enhancer Atlas [104]	Enhancer boundaries	Chromosomal positions for enhancer boundaries from all available tissue samples were downloaded in May 2018.	http://enhanceratlas.org/
Super Enhancer Archive [105]	Super-enhancer boundaries	Chromosomal positions for super enhancer boundaries from all available tissue samples were downloaded in May 2018.	http://sea.edbc.org/
ENCODE [106]	Histone ChIP-seq (p-value of peaks)	H3K4ME3, H3K4ME1, and H3K27AC ChIP-seq experiment bed files for all available cell lines were downloaded from the ENCODE experiment database in May 2018. All bed files were modified to contain the cell line, histone modification and peak p-value as a column entry.	www.encodeproject.org
ENCODE [1]	Cell Fraction Expression (proportion of total RPKM),	FPKMs for all transcripts in nuclear and cytoplasmic RNA-seq for GM12878 cells were downloaded in March 2018. The fraction of total reads in the nuclear fraction for each transcript was calculated.	www.encodeproject.org
ENCODE [107]	RNA-Binding Protein (eCLIP) (interacting partners, number RBP bound, number RBP binding sites),	eCLIP experiment bed files for replicates 01 and 02 from K562 and HepG2 cells for all available RBPs were downloaded from the ENCODE experiment database in May 2018. All bed files were modified to contain the RBP gene name, replicate number and cell line as column entries.	www.encodeproject.org
ENCODE [49]	ChIA-PET (boundaries of interacting fragments, score)	POL2RA ChIA-PET interactions from K562 and MCF-7 cells were downloaded in April 2018.	www.encodeproject.org
ENSEMBL BIOMART [108]	Gene Ontology, Transcript strand, Transcript Name	A biomart query was performed in May 2018. “Gene description”, “Strand”, and “Gene name” were selected and downloaded for all hg19 transcripts.	http://grch37.ensembl.org/biomart/martview/
Ren Lab Hi-C Project [40]	Topologically Associating Domain (TAD) Boundaries	Chromosomal positions were downloaded from two combined replicates of Human ES Cells.	http://chromosome.sdsc.edu/mouse/hi-c/download.html
RBP Image Database [82]	Sub-cellular localization of RNA Binding Proteins	Localization of all RBPs in HepG2 cells was downloaded and pruned to show only Nuclear and Cytoplasmic compartments.	http://rnabiology.ircm.qc.ca/RBPImage/

## Additional files

Additional file 1: **Figure S1.** Distribution of RNA types from de novo RNA analysis pipeline for primary NHL and normal B cell samples. A) Pie chart displays the percentage of total transcripts for each RNA category identified by the RNA-seq discovery pipeline. B) Violin plots show the range of expression (FPKM) in each category (solid dot = mean, vertical inner line = 25th - 75th interquartile range, tails = min - max values). FPKM = Fragments Per Kilobase of transcript per Million mapped reads. C) Bar plots show the cumulative percent of RNA transcripts in each category that were detected in the indicated percentage of samples. (PDF 1278 kb)

Additional file 2: **Table S1.** Detailed PLAIDOH output file columns. Contains descriptions of each column in the default “Output_” table created by PLAIDOH using either the provided Example input table or any user-provided input expression table that is properly formatted as described in Table 1. (XLSX 11 kb)

Additional file 3: **Figure S2.** LCP correlation, but not lncRNA expression, demonstrates an inverse relationship with distance for positively correlated LCPs. A&C) Box plots show lncRNA expression (FPKM, A) or LCP correlation (−log10 Spearman adjusted *p*-value, C) binned by distance to individual coding genes within 400 kb flanking the lncRNA (top panel: positively correlated LCPs, bottom panel: negatively correlated LCPs). B&D) Box plots show lncRNA expression (FPKM, B) or LCP correlation (−log10 Spearman adjusted p-value, D) for four categories of lncRNA. (PDF 1069 kb)

Additional file 4: **Figure S3.** PLAIDOH reveals a landscape of enhancer regulatory activity and LCP co-expression. A) Box plots show the number of lncRNAs overlapping conventional or super enhancers per kb. B) Box plots show the expression of lncRNAs in conventional or super enhancers, or not overlapping any enhancer. C) Box plots show the expression of lncRNAs that do or do not overlap coding genes (intra- or inter-genic, pink or purple, respectively), and coincide with conventional or super enhancers, or no enhancer. * *p* < 0.05, ** *p* < 0.01, *** *p* < 0.001, **** *p* < 0.0001. D) LncRNA expression (log10 FPKM) or E) LCP correlation (−log 10 Spearman adjusted p-value) relative to the intensity of activating and/or enhancer-associated histone marks (−log10 p-value for peaks). Red lines and grey zones indicate the regression lines and confidence intervals, respectively. (PDF 995 kb)

Additional file 5: **Figure S4.** LncRNA--coding gene interaction frequency and nuclear localization are associated with higher and more significant correlation coefficients. A) Histograms show the number of LCPs that have CHIA interactions (positive, blue) or no CHIA interactions (negative, red) binned by distance to the coding gene. B) Histogram shows the number of LCPs binned by distance to the coding gene. Regression line traces the CHIA score for LCPs in each bin. C) Histograms show the number of LCPs binned by distance to the coding gene that have significant (adjusted *p* < 0.05, blue) or non-significant (adjusted *p* > 0.05, red) Spearman expression correlation coefficients. D) Box plot shows the absolute Spearman correlation of negatively (left, black) or positively (right, blue) correlated LCPs, binned by fraction nuclear localization of the lncRNA. (PDF 2328 kb)

Additional file 6: **Figure S5.** LncRNAs demonstrate common or cancer-type specific positive or negative correlation profiles. A) Venn diagrams show the number of significant LCPs shared or unique among five TCGA cancer types for positively (left) or negatively (right) correlated pairs. Significant = Spearman correlation adj p < 0.05 for LCP expression. B) Binary heatmaps show the pattern of correlation significance for LCPs across TCGA cancer types for positively (left) or negatively (right) correlated pairs. Spearman adj *p* < 0.05 (purple); *p* > 0.05 (white). C) Bar graph shows the expression of coding genes within +/− 400 kb of *AC096992.2* in TCGA cancer types. D) Bar graph shows the levels of the indicated histone marks by ChIP-seq for the region near *AC096992.2*. No peaks were detected in BRCA/MCF7 and DLBC/OCI-LY7. (PDF 1453 kb)

Additional file 7: **Figure S6.** PLAIDOH ranks LCPs by Enhancer and LncRNA *Cis-*regulatory scores to predict likely transcriptional regulatory mechanism. A-L) Plots show LCPs from TCGA cancers (AML A-C, BRCA D-F, CESC G-I, LUAD J-L). Plotted as in Fig. 6. (A,D,G & J) Plots show LCPs ranked by increasing LncRNA Transcript *Cis-*regulatory Scores. Highlighted in red are known *cis-*acting lncRNAs; in green are novel LCPs with the highest scores. B,E,H & K) As in A,D,G,J, but ranked by increasing Enhancer Scores. Red points are known enhancer-associated lncRNAs; in green are novel LCPs with the highest scores. C,F,I & L) Plots show Enhancer versus LncRNA Transcript *Cis-*regulatory Scores segregating LCPs by relative likelihood of each transcriptional regulatory mechanism. Dotted lines in A-L reflect score cut-offs based on the geometric inflection points calculated from the data in A, B, D, E, G, H, J, and K. Red and green data points are from A&B (for C), D&E (for F), G&H (for I), J&K (for L). (PDF 9087 kb)

Additional file 8: **Figure S7.** PLAIDOH Compares Favorably to lncRNA CRISPR Screens and to Other lncRNA Analytical Tools. A) Box plot shows Enhancer and lncRNA Transcript cis-regulatory scores in hits compared to non-hits in a CRISPR-KRAB lncRNA screen [28]. B) XY plot shows Enhancer versus LncRNA Transcript *Cis-*regulatory Scores segregating LCPs in ENCODE cell lines, plotted as in Fig. 6C. Red points are validated hits from a lncRNA knock-out (splice-site targeted) CRISPR screen [78]. C) XY plot shows Enhancer versus LncRNA Transcript *Cis-*regulatory Scores segregating LCPs in AML TCGA samples, plotted as in Fig. S6C. Red points are validated hits from a lncRNA CRISPR-activating screen [79]. D) XY plot shows Enhancer versus LncRNA Transcript *Cis-*regulatory Scores segregating LCPs ENCODE cell lines, plotted as in Fig. 6C. Colored data points reflect predicted function of highly ranked lncRNAs from [22]. Red circles indicate lncRNAs that are predicted to act at the transcriptional level, orange squares - post-transcriptional, green squares - both transcriptional and post-transcriptional. (PDF 2263 kb)

Additional file 9: **Figure S8.** PLAIDOH highly ranks lncRNA-RNA binding protein interactions from a lncRNA CRISPR Screen. Plot shows lncRNA expression versus RBP binding-site density per kilobase of RNA transcript for each lncRNA/RBP interaction, plotted as in Fig. 7B. Data point size is scaled to RBP expression level and subcellular localization interactions are colored as in Fig. 7B. Labeled dots highlight 3 validated hits from the CRISPR-KRAB growth screen [28] that had low PLAIDOH Enhancer and LncRNA transcript cis-regulatory scores (Fig. S7B). (PDF 2607 kb)

Additional file 10: **Figure S9.** Validation of PLAIDOH’s functional predictions for lncRNA RP11-960 L18.1 A) Expression of *PLCG2* and *RP11-960 L18.1* measured by qRT-PCR in control or in three independent *RP11-960 L18.1* KO subclones (U2932 lymphoma B cell line). B) Western Blot of PLCG2 or GAPDH in control (WT) or *RP11-960 L18.1* KO U2932 cells. Triangles indicate relative number of cells loaded on the gel. (PDF 2055 kb)

## Abbreviations

AML: Acute Myeloid Leukemia
BRCA: Breast Cancer
CESC: Cervical Squamous Cell Carcinoma
ChIA-PET: Chromatin Interaction Analysis by Paired-End Tag Sequencing
ChIP-seq: Chromatin Immunoprecipitation sequencing
CRISPRi: CRISPR Interference
DLBC: Diffuse Large B cell Lymphoma
eCLIP: Enhanced Crosslinking and Immunoprecipitation
ENCODE: Encyclopedia of DNA Elements
FANTOM: Functional ANnoTation Of the Mammalian genome
FPKM: Fragments Per Kilobase per Million reads
LCP: LncRNA and Coding-gene Pair
lncRNA: Long Non-coding RNA
LUAD: Lung Adenocarcinoma
NHL: Non-Hodgkin Lymphoma
PLAIDOH: Predicting LncRNA Activity through Integrative Data-driven ‘Omics and Heuristics
RBP: RNA Binding Protein
SE: Super Enhancer
TAD: Topologically Associating Domain
TCGA: The Cancer Genome Atlas
