# Supplementary figures and images for: PLAIDOH: a novel method for functional prediction of long non-coding RNAs identifies cancer-specific LncRNA activities
Source: BMC Genomics. 2019 Feb 15;20:137. doi: 10.1186/s12864-019-5497-4 (PMC6377765; doi:10.1186/s12864-019-5497-4)

**A**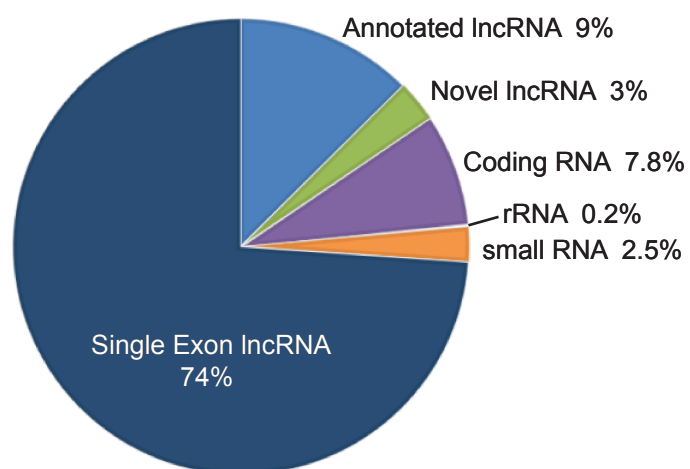**B**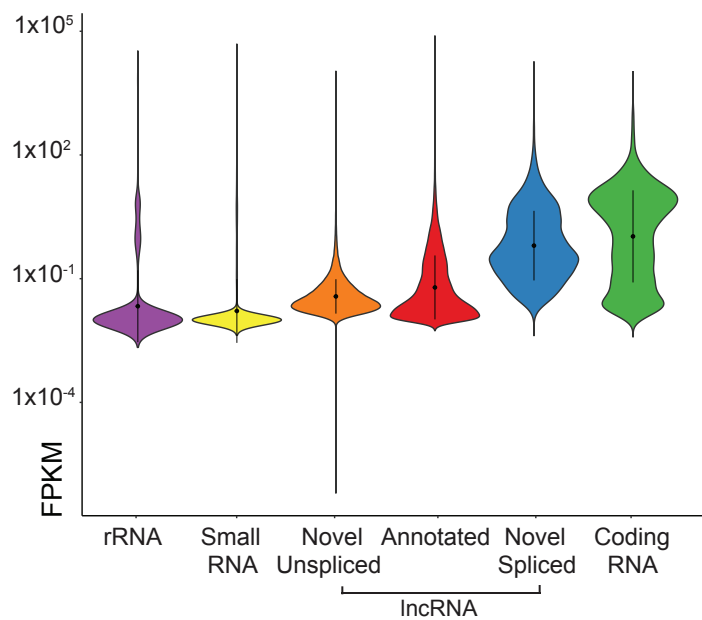**C**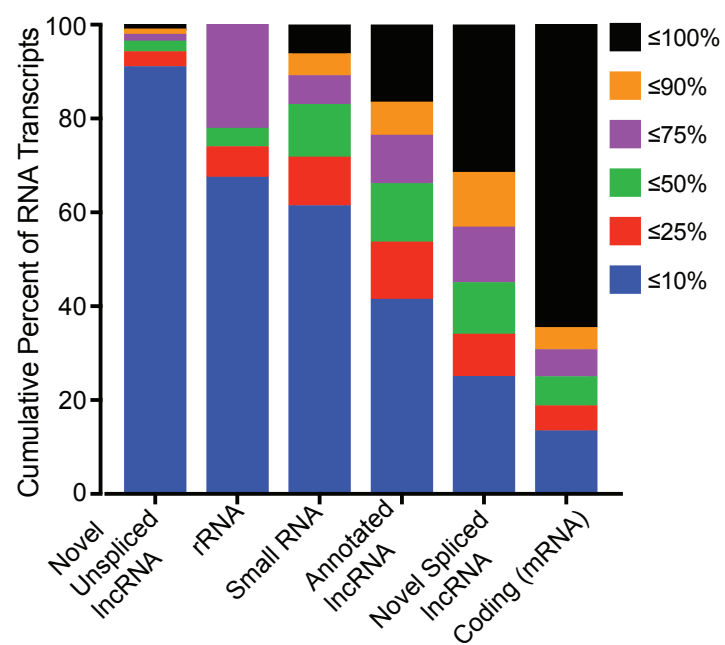

Supplement: Supplementary file 1 — Figure S1. Distribution of RNA types from de novo RNA analysis pipeline for primary NHL and normal B cell samples. A) Pie chart displays the percentage of total transcripts for each RNA category identified by the RNA-seq discovery pipeline. B) Violin plots show the range of expression (FPKM) in each category (solid dot = mean, vertical inner line = 25th - 75th interquartile range, tails = min - max values). FPKM = Fragments Per Kilobase of transcript per Million mapped reads. C) Bar plots show the cumulative percent of RNA transcripts in each category that were detected in the indicated percentage of samples. (PDF 1278 kb) [file 12864_2019_5497_MOESM1_ESM.pdf]

**A**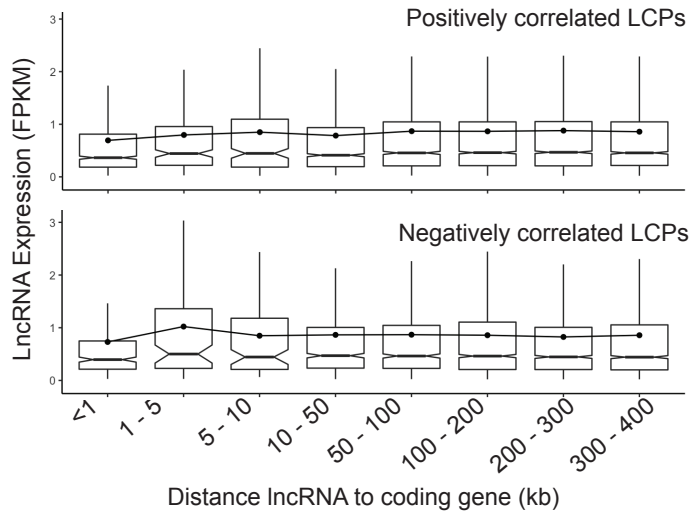**B**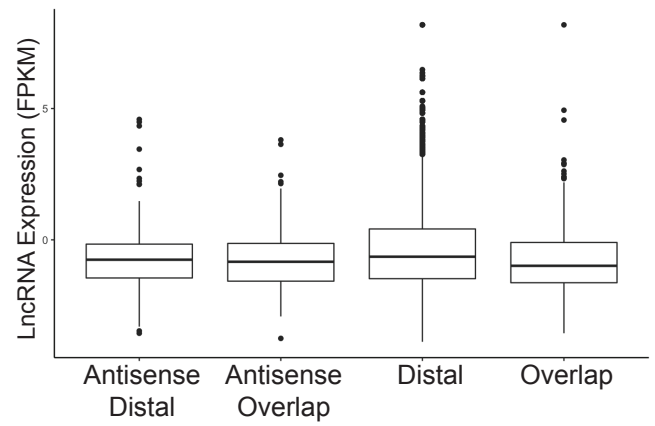**C**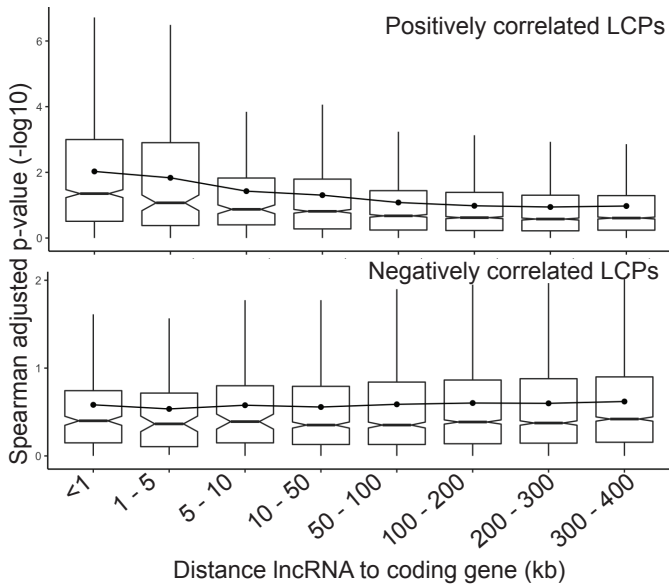**D**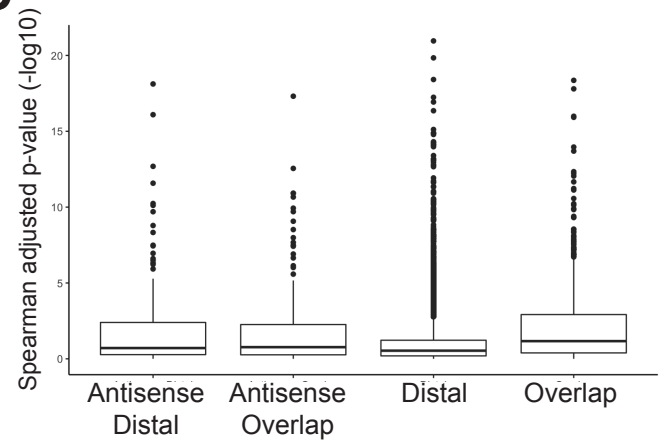

Supplement: Supplementary file 3 — Figure S2. LCP correlation, but not lncRNA expression, demonstrates an inverse relationship with distance for positively correlated LCPs. A&C) Box plots show lncRNA expression (FPKM, A) or LCP correlation (−log10 Spearman adjusted p-value, C) binned by distance to individual coding genes within 400 kb flanking the lncRNA (top panel: positively correlated LCPs, bottom panel: negatively correlated LCPs). B&D) Box plots show lncRNA expression (FPKM, B) or LCP correlation (−log10 Spearman adjusted p-value, D) for four categories of lncRNA. (PDF 1069 kb) [file 12864_2019_5497_MOESM3_ESM.pdf]

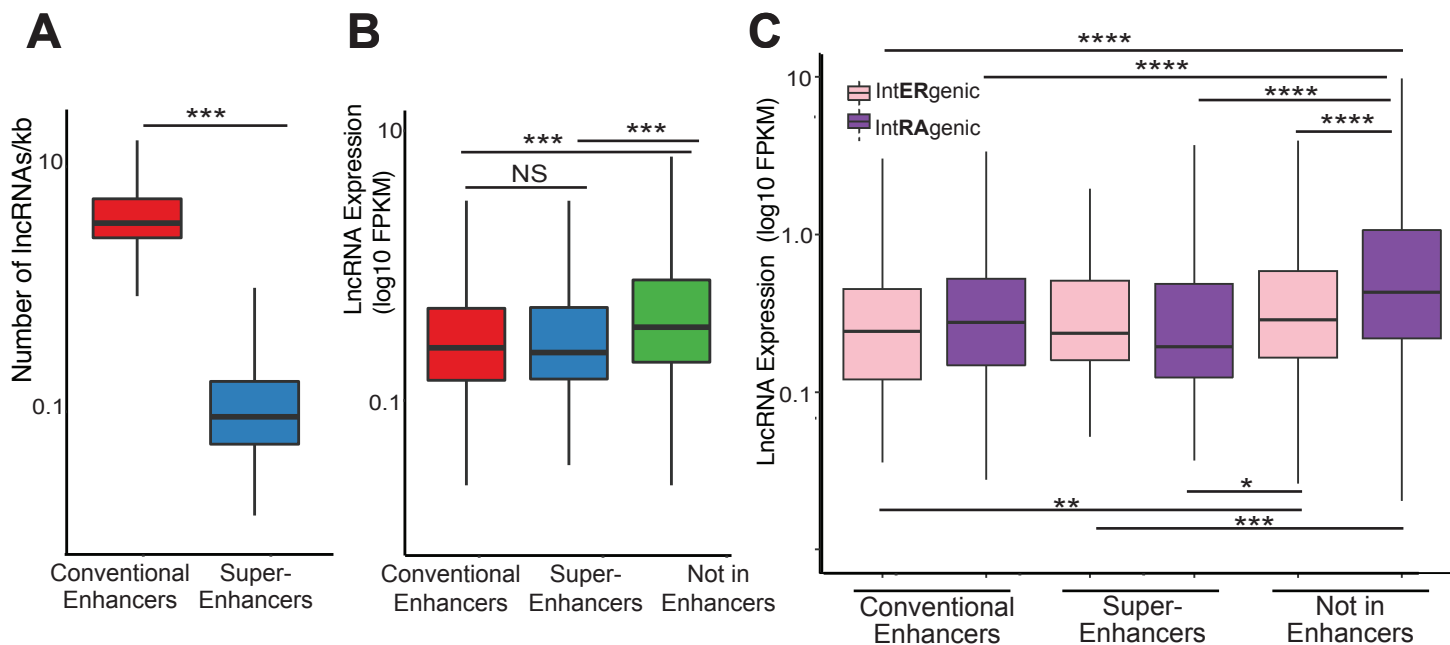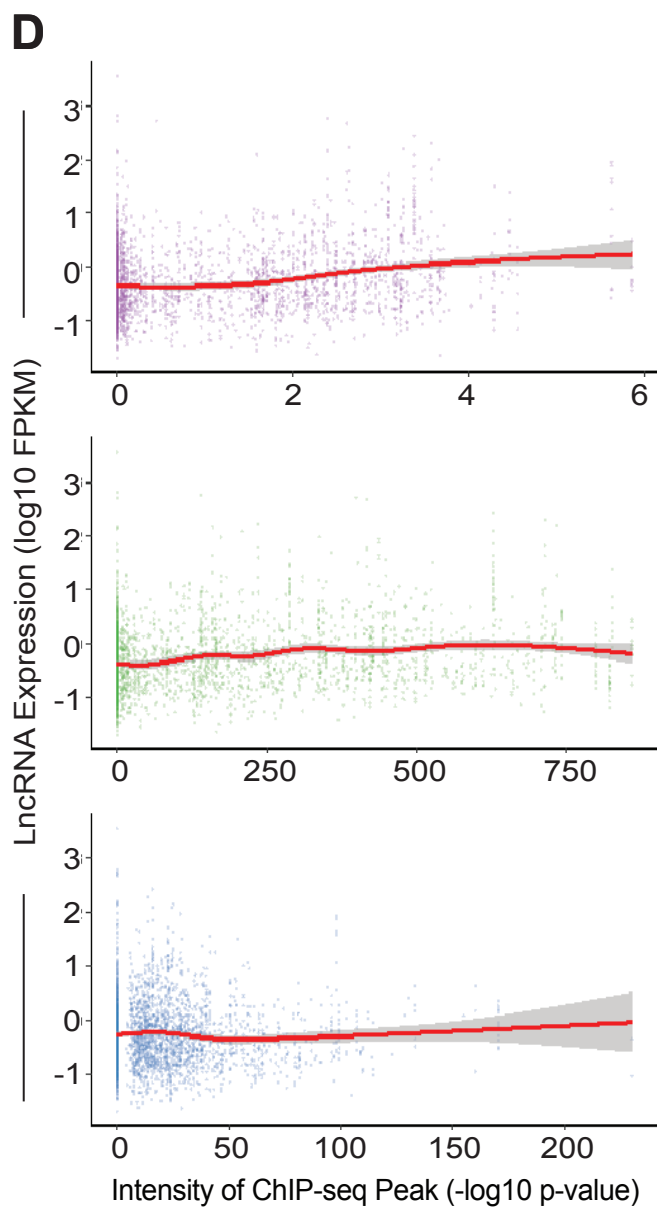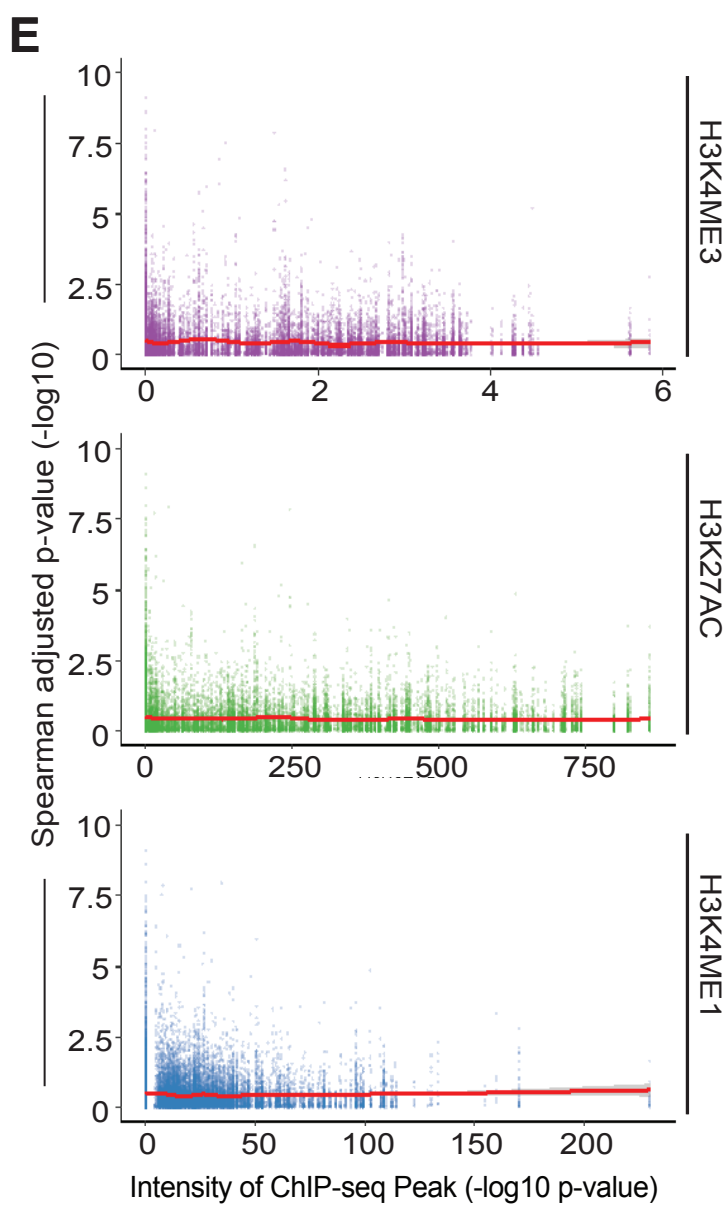

Supplement: Supplementary file 4 — Figure S3. PLAIDOH reveals a landscape of enhancer regulatory activity and LCP co-expression. A) Box plots show the number of lncRNAs overlapping conventional or super enhancers per kb. B) Box plots show the expression of lncRNAs in conventional or super enhancers, or not overlapping any enhancer. C) Box plots show the expression of lncRNAs that do or do not overlap coding genes (intra- or inter-genic, pink or purple, respectively), and coincide with conventional or super enhancers, or no enhancer. * p < 0.05, ** p < 0.01, *** p < 0.001, **** p < 0.0001. D) LncRNA expression (log10 FPKM) or E) LCP correlation (−log 10 Spearman adjusted p-value) relative to the intensity of activating and/or enhancer-associated histone marks (−log10 p-value for peaks). Red lines and grey zones indicate the regression lines and confidence intervals, respectively. (PDF 995 kb) [file 12864_2019_5497_MOESM4_ESM.pdf]

**A**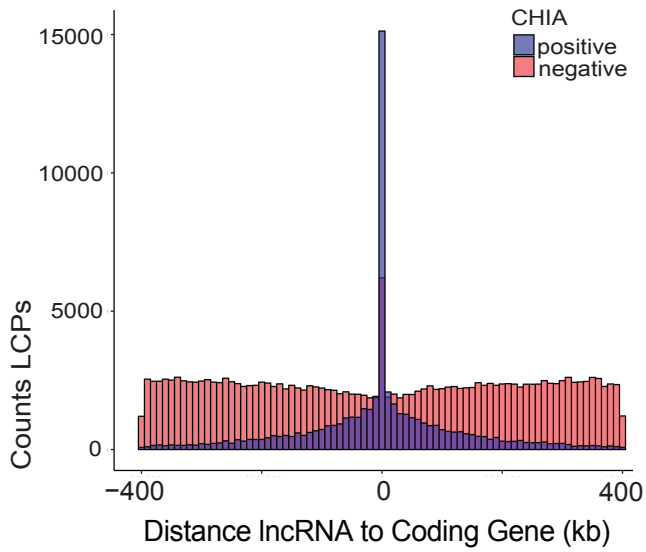**B**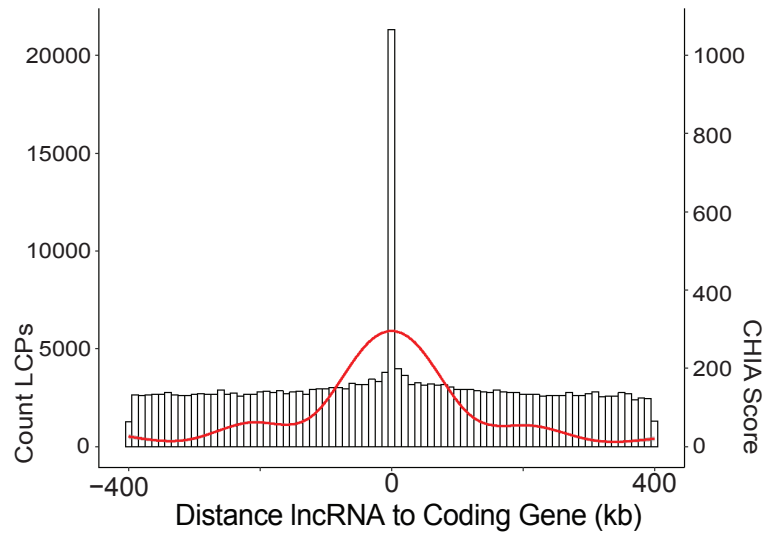**C**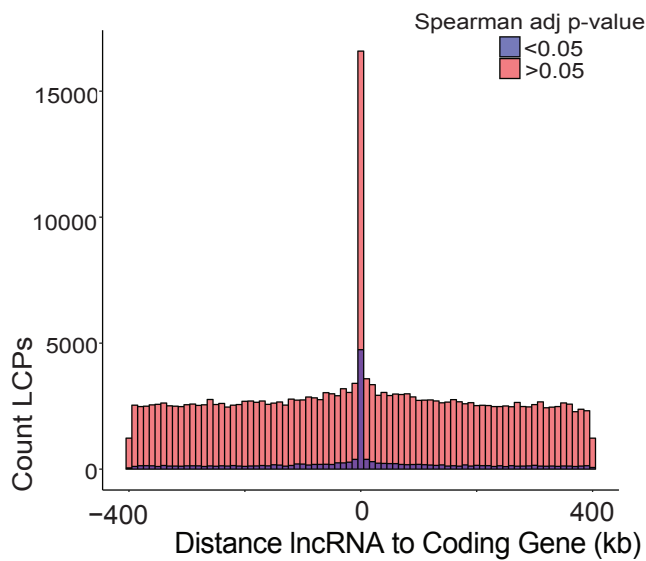**D**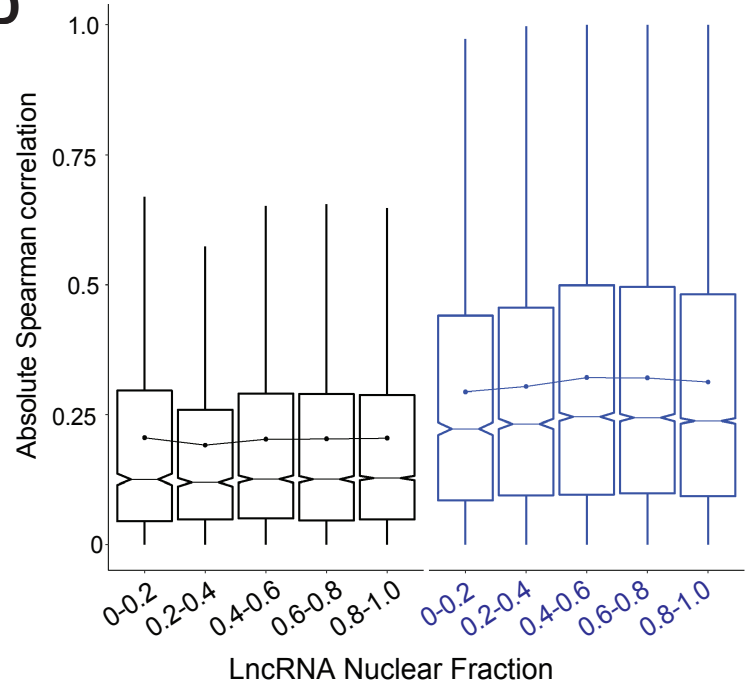

Supplement: Supplementary file 5 — Figure S4. LncRNA--coding gene interaction frequency and nuclear localization are associated with higher and more significant correlation coefficients. A) Histograms show the number of LCPs that have CHIA interactions (positive, blue) or no CHIA interactions (negative, red) binned by distance to the coding gene. B) Histogram shows the number of LCPs binned by distance to the coding gene. Regression line traces the CHIA score for LCPs in each bin. C) Histograms show the number of LCPs binned by distance to the coding gene that have significant (adjusted p < 0.05, blue) or non-significant (adjusted p > 0.05, red) Spearman expression correlation coefficients. D) Box plot shows the absolute Spearman correlation of negatively (left, black) or positively (right, blue) correlated LCPs, binned by fraction nuclear localization of the lncRNA. (PDF 2328 kb) [file 12864_2019_5497_MOESM5_ESM.pdf]

**A**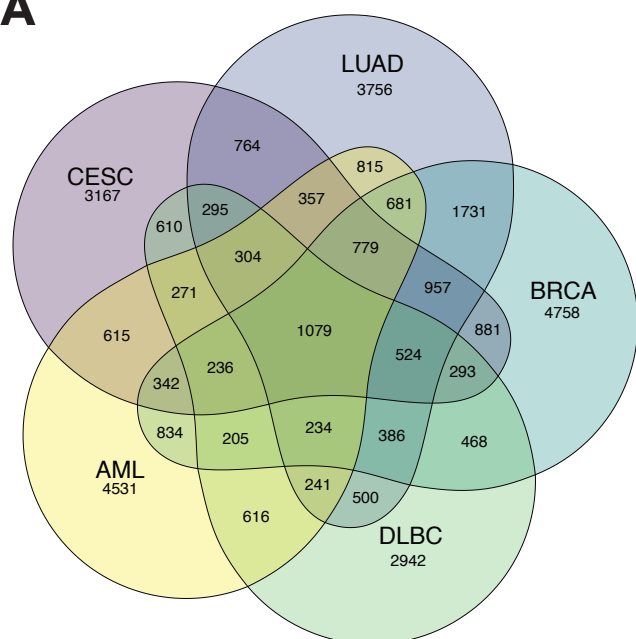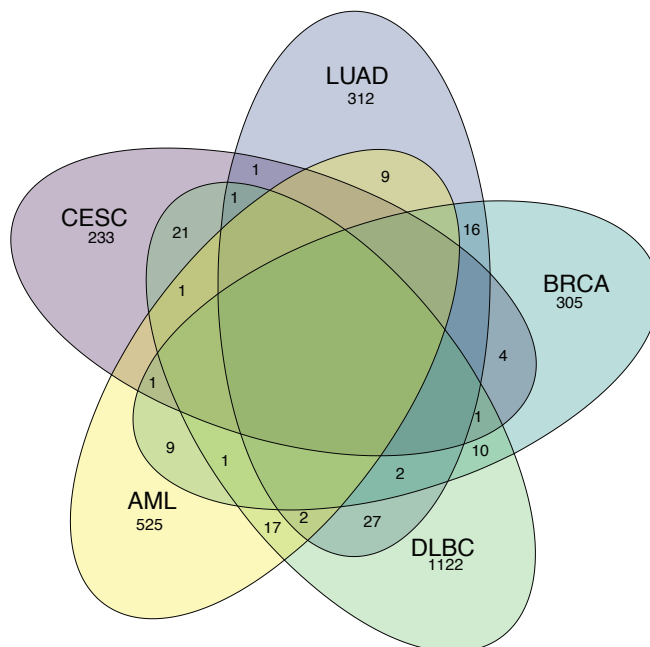**B**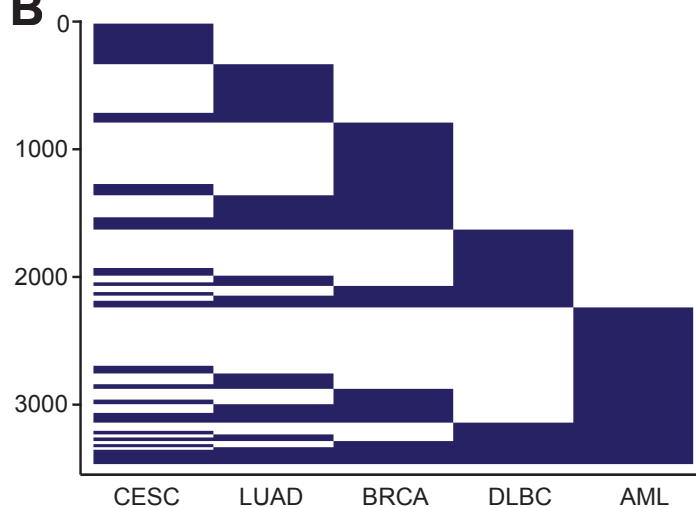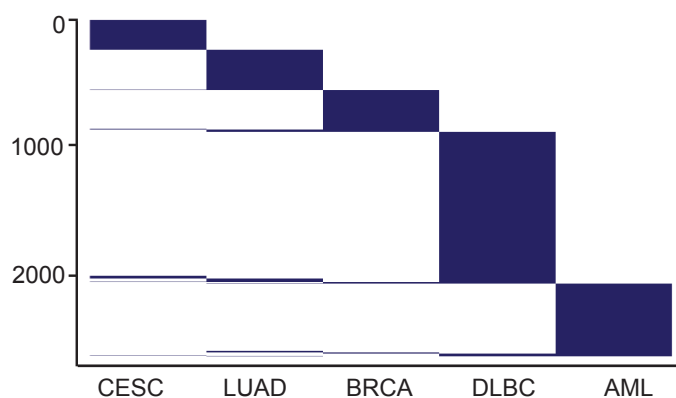**C**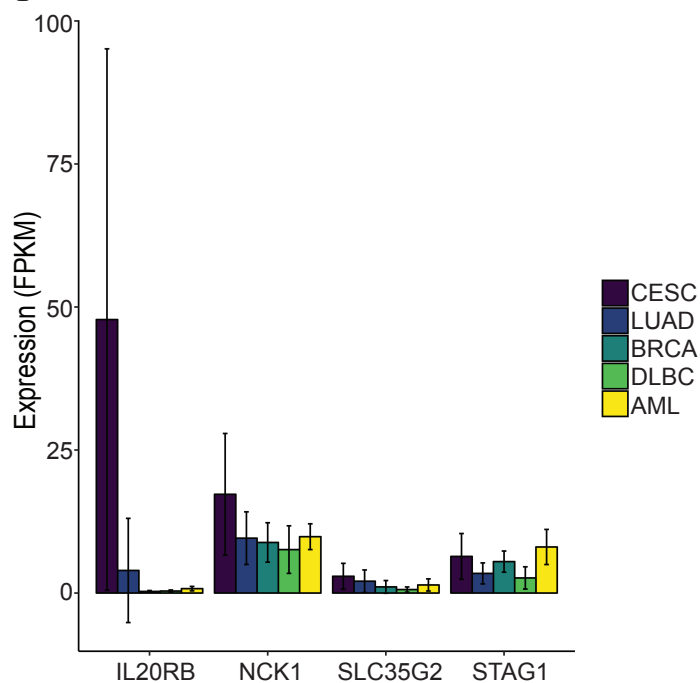**D**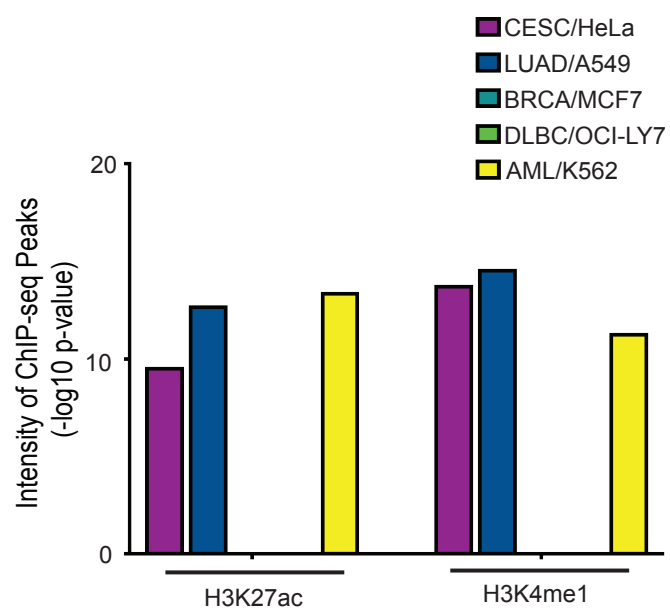

Supplement: Supplementary file 6 — Figure S5. LncRNAs demonstrate common or cancer-type specific positive or negative correlation profiles. A) Venn diagrams show the number of significant LCPs shared or unique among five TCGA cancer types for positively (left) or negatively (right) correlated pairs. Significant = Spearman correlation adj p < 0.05 for LCP expression. B) Binary heatmaps show the pattern of correlation significance for LCPs across TCGA cancer types for positively (left) or negatively (right) correlated pairs. Spearman adj p < 0.05 (purple); p > 0.05 (white). C) Bar graph shows the expression of coding genes within +/− 400 kb of AC096992.2 in TCGA cancer types. D) Bar graph shows the levels of the indicated histone marks by ChIP-seq for the region near AC096992.2. No peaks were detected in BRCA/MCF7 and DLBC/OCI-LY7. (PDF 1453 kb) [file 12864_2019_5497_MOESM6_ESM.pdf]

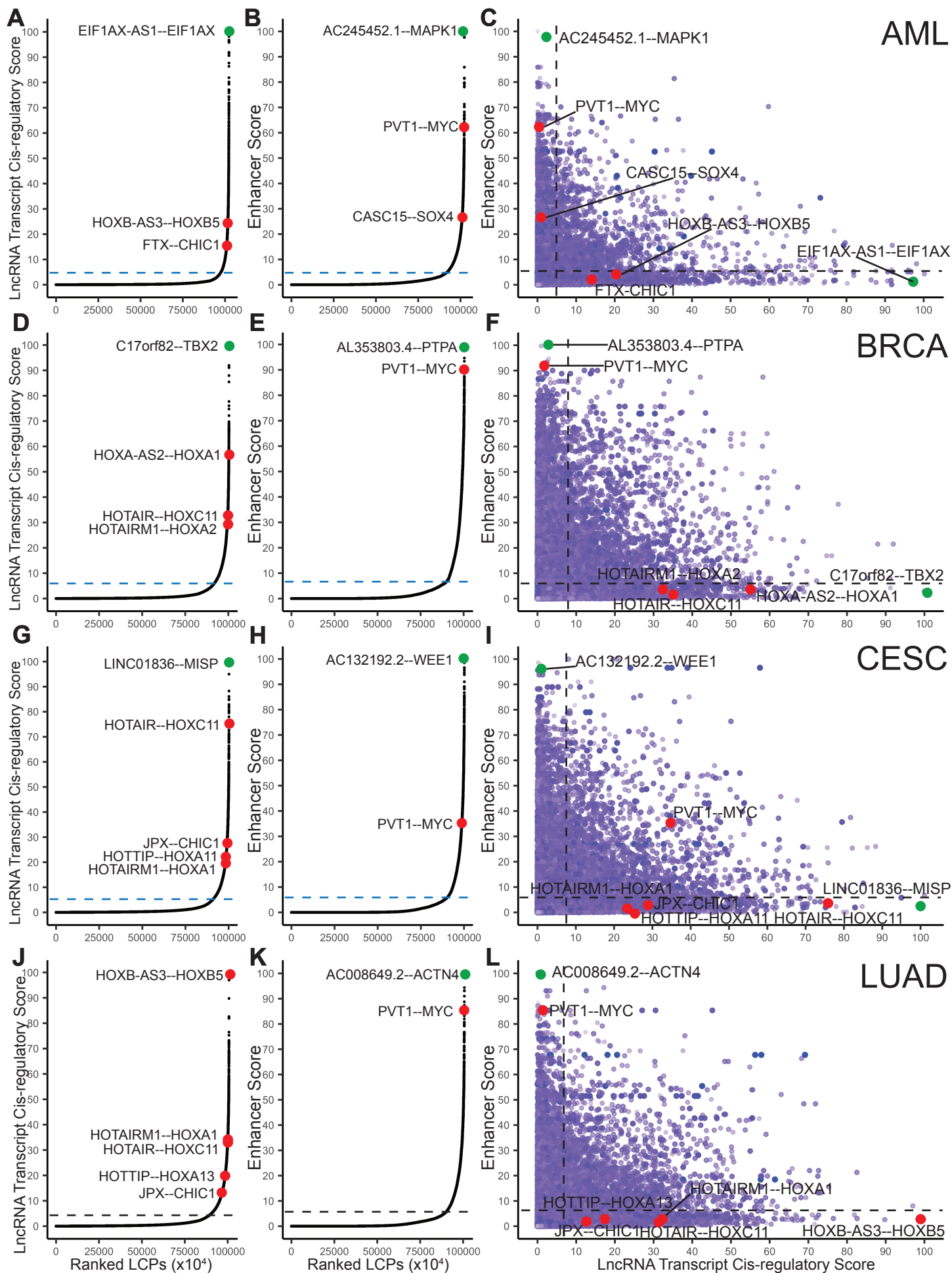

Supplement: Supplementary file 7 — Figure S6. PLAIDOH ranks LCPs by Enhancer and LncRNA Cis-regulatory scores to predict likely transcriptional regulatory mechanism. A-L) Plots show LCPs from TCGA cancers (AML A-C, BRCA D-F, CESC G-I, LUAD J-L). Plotted as in Fig. 6. (A,D,G & J) Plots show LCPs ranked by increasing LncRNA Transcript Cis-regulatory Scores. Highlighted in red are known cis-acting lncRNAs; in green are novel LCPs with the highest scores. B,E,H & K) As in A,D,G,J, but ranked by increasing Enhancer Scores. Red points are known enhancer-associated lncRNAs; in green are novel LCPs with the highest scores. C,F,I & L) Plots show Enhancer versus LncRNA Transcript Cis-regulatory Scores segregating LCPs by relative likelihood of each transcriptional regulatory mechanism. Dotted lines in A-L reflect score cut-offs based on the geometric inflection points calculated from the data in A, B, D, E, G, H, J, and K. Red and green data points are from A&B (for C), D&E (for F), G&H (for I), J&K (for L). (PDF 9087 kb) [file 12864_2019_5497_MOESM7_ESM.pdf]

**A**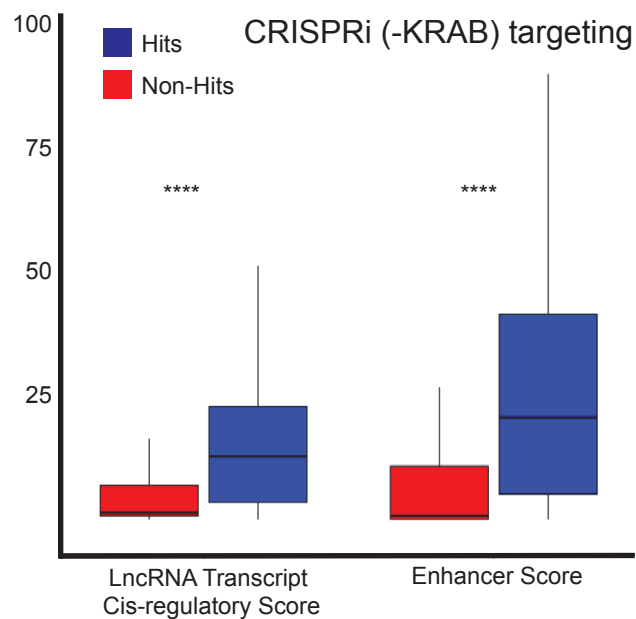**B**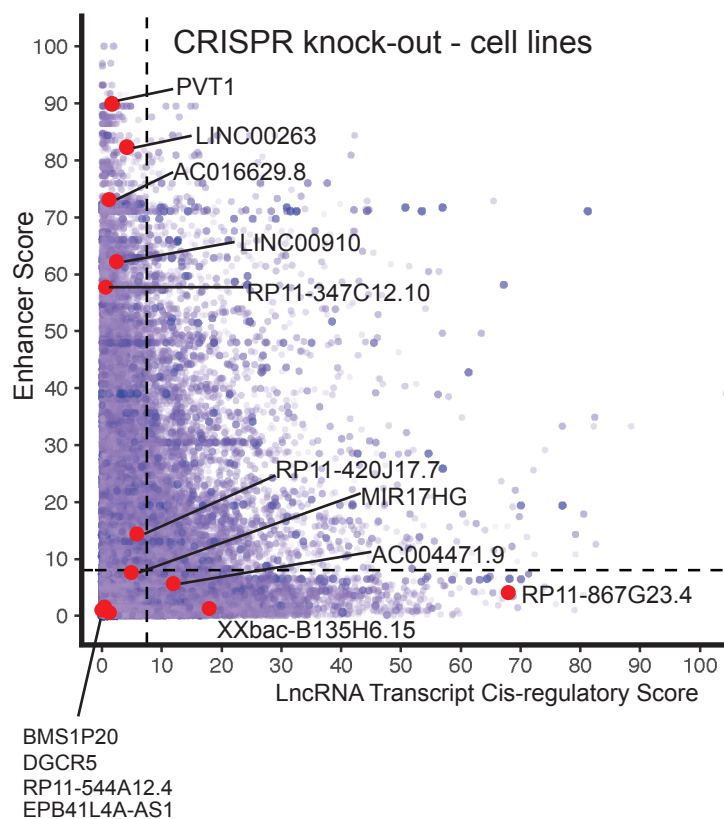**C**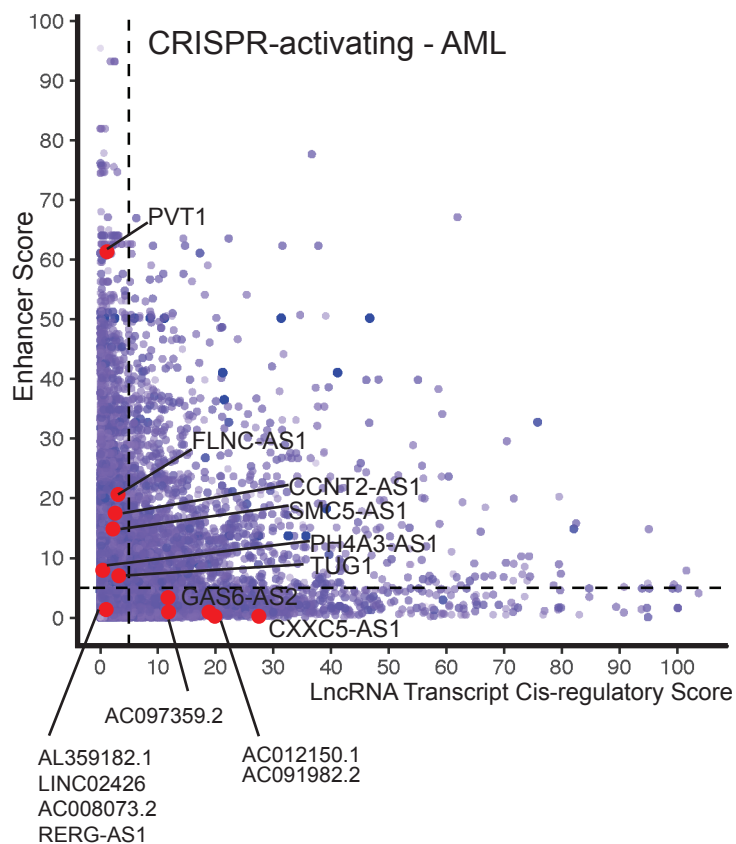**D**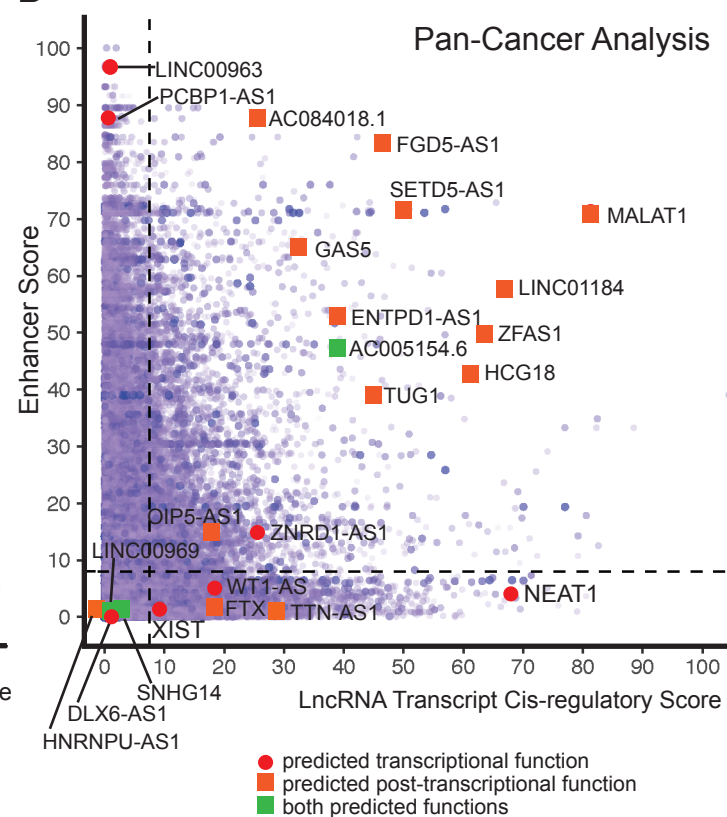

Supplement: Supplementary file 8 — Figure S7. PLAIDOH Compares Favorably to lncRNA CRISPR Screens and to Other lncRNA Analytical Tools. A) Box plot shows Enhancer and lncRNA Transcript cis-regulatory scores in hits compared to non-hits in a CRISPR-KRAB lncRNA screen [28]. B) XY plot shows Enhancer versus LncRNA Transcript Cis-regulatory Scores segregating LCPs in ENCODE cell lines, plotted as in Fig. 6C. Red points are validated hits from a lncRNA knock-out (splice-site targeted) CRISPR screen [78]. C) XY plot shows Enhancer versus LncRNA Transcript Cis-regulatory Scores segregating LCPs in AML TCGA samples, plotted as in Fig. S6C. Red points are validated hits from a lncRNA CRISPR-activating screen [79]. D) XY plot shows Enhancer versus LncRNA Transcript Cis-regulatory Scores segregating LCPs ENCODE cell lines, plotted as in Fig. 6C. Colored data points reflect predicted function of highly ranked lncRNAs from [22]. Red circles indicate lncRNAs that are predicted to act at the transcriptional level, orange squares - post-transcriptional, green squares - both transcriptional and post-transcriptional. (PDF 2263 kb) [file 12864_2019_5497_MOESM8_ESM.pdf]

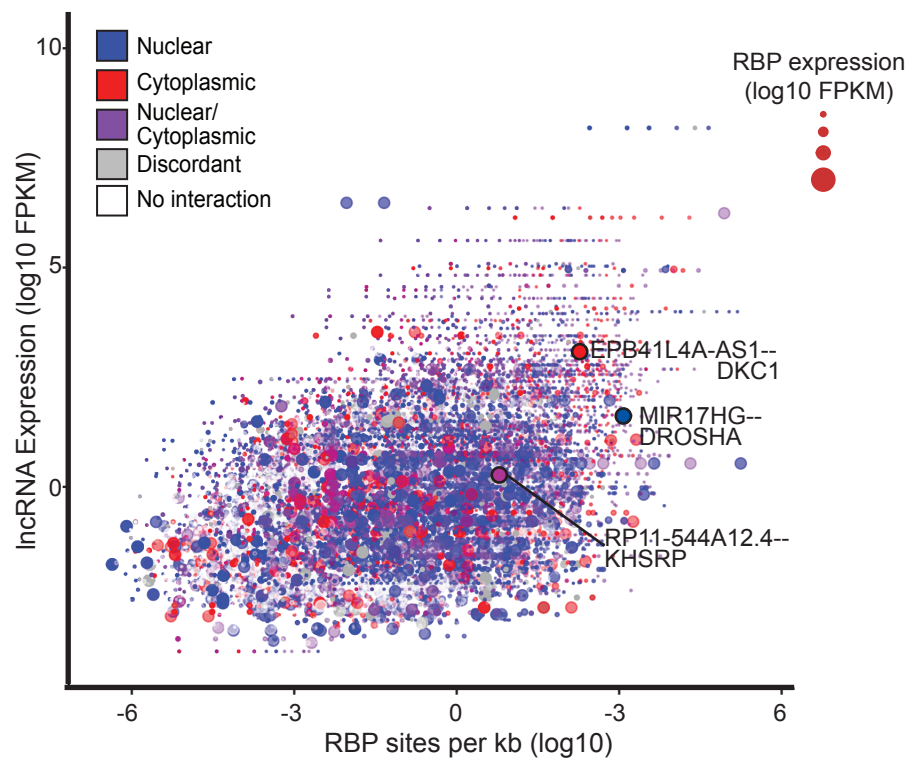

Supplement: Supplementary file 9 — Figure S8. PLAIDOH highly ranks lncRNA-RNA binding protein interactions from a lncRNA CRISPR Screen. Plot shows lncRNA expression versus RBP binding-site density per kilobase of RNA transcript for each lncRNA/RBP interaction, plotted as in Fig. 7B. Data point size is scaled to RBP expression level and subcellular localization interactions are colored as in Fig. 7B. Labeled dots highlight 3 validated hits from the CRISPR-KRAB growth screen [28] that had low PLAIDOH Enhancer and LncRNA transcript cis-regulatory scores (Fig. S7B). (PDF 2607 kb) [file 12864_2019_5497_MOESM9_ESM.pdf]

**A**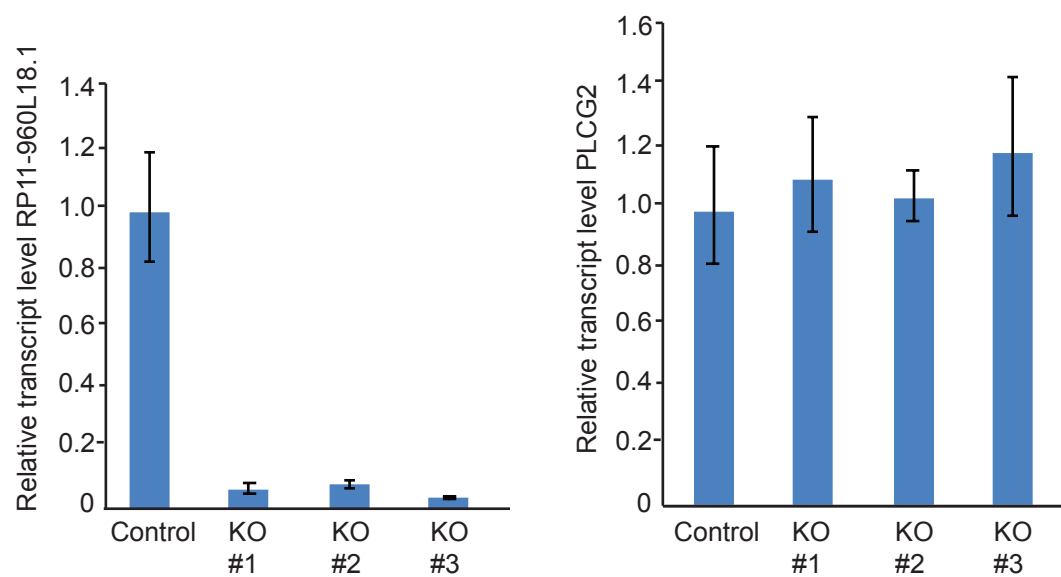**B**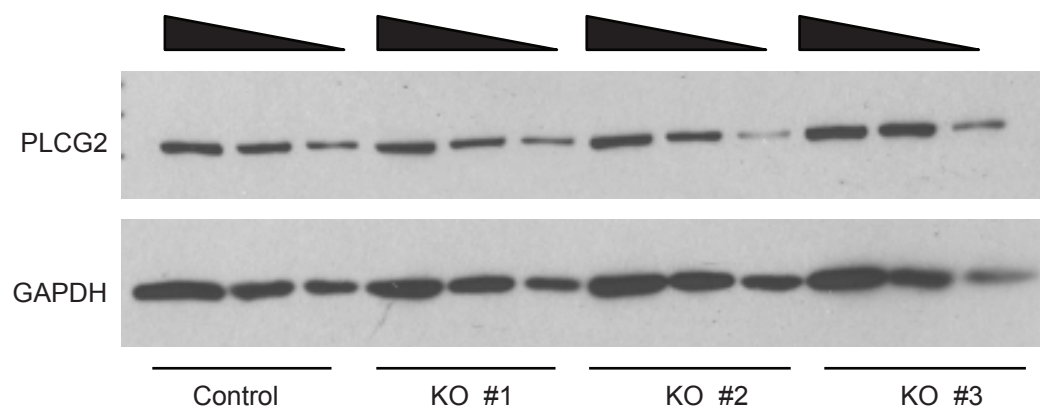

Supplement: Supplementary file 10 — Figure S9. Validation of PLAIDOH’s functional predictions for lncRNA RP11-960 L18.1 A) Expression of PLCG2 and RP11-960 L18.1 measured by qRT-PCR in control or in three independent RP11-960 L18.1 KO subclones (U2932 lymphoma B cell line). B) Western Blot of PLCG2 or GAPDH in control (WT) or RP11-960 L18.1 KO U2932 cells. Triangles indicate relative number of cells loaded on the gel. (PDF 2055 kb) [file 12864_2019_5497_MOESM10_ESM.pdf]
